# Stingless bee propolis: a comprehensive review of chemical constituents and health efficacy

**DOI:** 10.1007/s13659-025-00545-4

**Published:** 2025-09-04

**Authors:** Nosiba A. Alsarayrah, Rafeezul Mohamed, Eshaifol A. Omar

**Affiliations:** 1 Department of Toxicology, Advanced Medical and Dental Institute, Universiti Sains Malaysia, Bertam, 13200 Kepala Batas, Pulau Pinang Malaysia; 2 Department of Biomedical Science, Advanced Medical and Dental Institute, Universiti Sains Malaysia, Kepala Batas, Pulau Pinang Malaysia

**Keywords:** Antioxidant, Nutritional supplements, Chemical analysis, Bioactive constituents, Propolis, Stingless bee

## Abstract

Propolis, consisting of plant-derived materials, wax, and bee secretions, is abundant in bioactive constituents like flavonoids, phenolic compounds, and terpenes, which enhance its various biological functions. These encompass antioxidant, anti-inflammatory, antibacterial, anticancer, antidiabetic, and immunomodulatory properties. Propolis has demonstrated effectiveness in the prevention and treatment of multiple illnesses, including cardiovascular disease, atherosclerosis, infections, diabetes, wound healing, and burns. Its extensive health benefits endorse its application in medications, nutritional supplements, and cosmetics, where it is acknowledged as a safe and efficacious natural product. Propolis, whether utilized in its raw state, as extracts, or in conjunction with other products, exhibits considerable promise in alternative medicine and nutritional health. Propolis extracts are crucial to examine as a key component in health and wellness, offering prospective applications in disease prevention and therapeutic support Further research is necessary to clarify its molecular mechanisms, examine potential allergic reactions, and determine ideal dosages for various ages. This article provides a comprehensive comparative examination of various propolis types, emphasizing their distinct phytochemical contents and varying biological effects concurrently. It integrates results from both in vitro and in vivo investigations, enhancing the comprehension of health applications and mechanisms of action, grounded comparisons in pertinent prior studies.

**Graphical Abstract:**

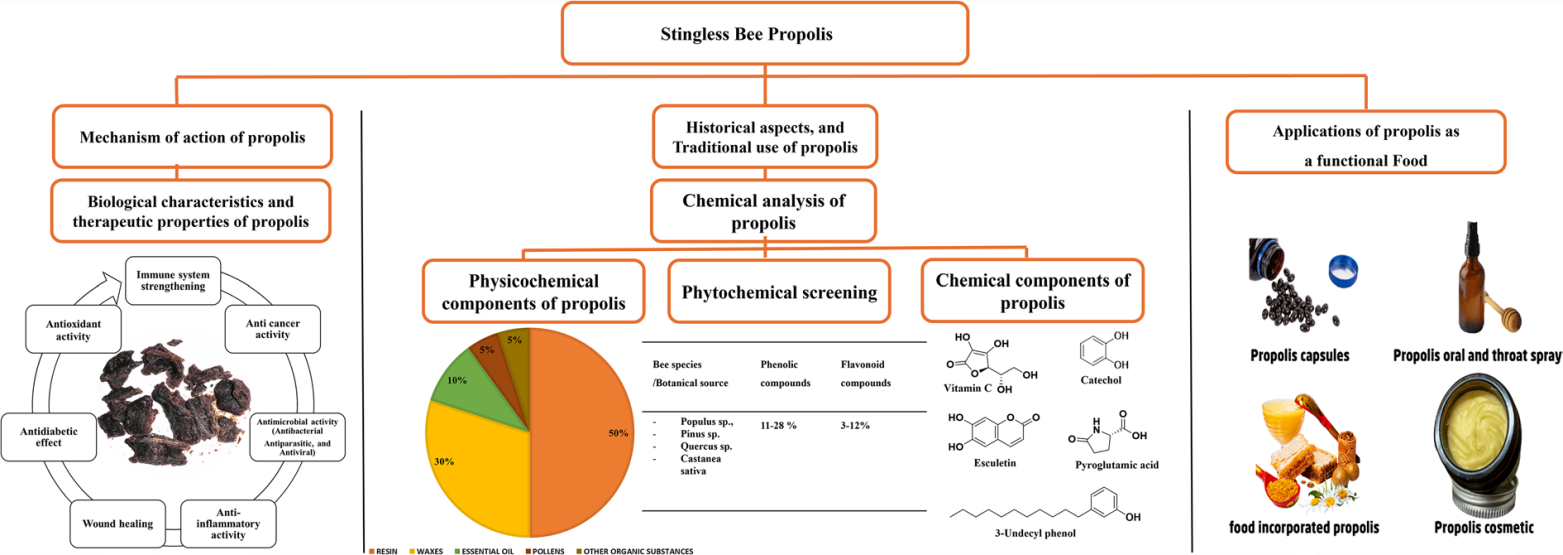

**Supplementary Information:**

The online version contains supplementary material available at 10.1007/s13659-025-00545-4.

## Methods

### Search strategy

The search strategy focused on literature directly addressing the primary topic, while studies with less relevant content were excluded. Key electronic databases, search engines, Web of Science, Google Scholar, and ScienceDirect for articles meeting the inclusion standards.

The search involved specific keyword combinations and Medical Subject Headings (MESH) in titles and abstracts, using terms such as: (Phytochemical, antioxidant, anti-cancer, bioactivity, inflammation, cardiovascular disease, antimicrobial, diabetes, wound healing, preclinical studies, and clinical studies), alongside non-MESH (bee propolis, phytochemicals, free radicals, stingless bee propolis). Search was refined with the “AND” operator to enhance relevance. This strategy yielded a broad selection of pertinent articles.

### Inclusion criteria

Open-access publications from academic search engines were selected based on their direct relevance to the research topic and their ability to provide accurate, comprehensive, and up-to-date data. The inclusion criteria targeted observational and experimental studies examining the origins of propolis, its traditional uses, phytochemical composition, and biological efficacy, including both in vitro and animal studies. Non-original publications (e.g., reviews, letters, or comments), redundant studies, and those lacking full-text availability or addressing unrelated conditions were excluded. Only studies published in English were considered, and authorship rights were respected through proper citation. This systematic and selective approach ensured that the analysis was grounded in credible scientific evidence and aligned with ethical research standards.

## Introduction

**Fig. 1:**
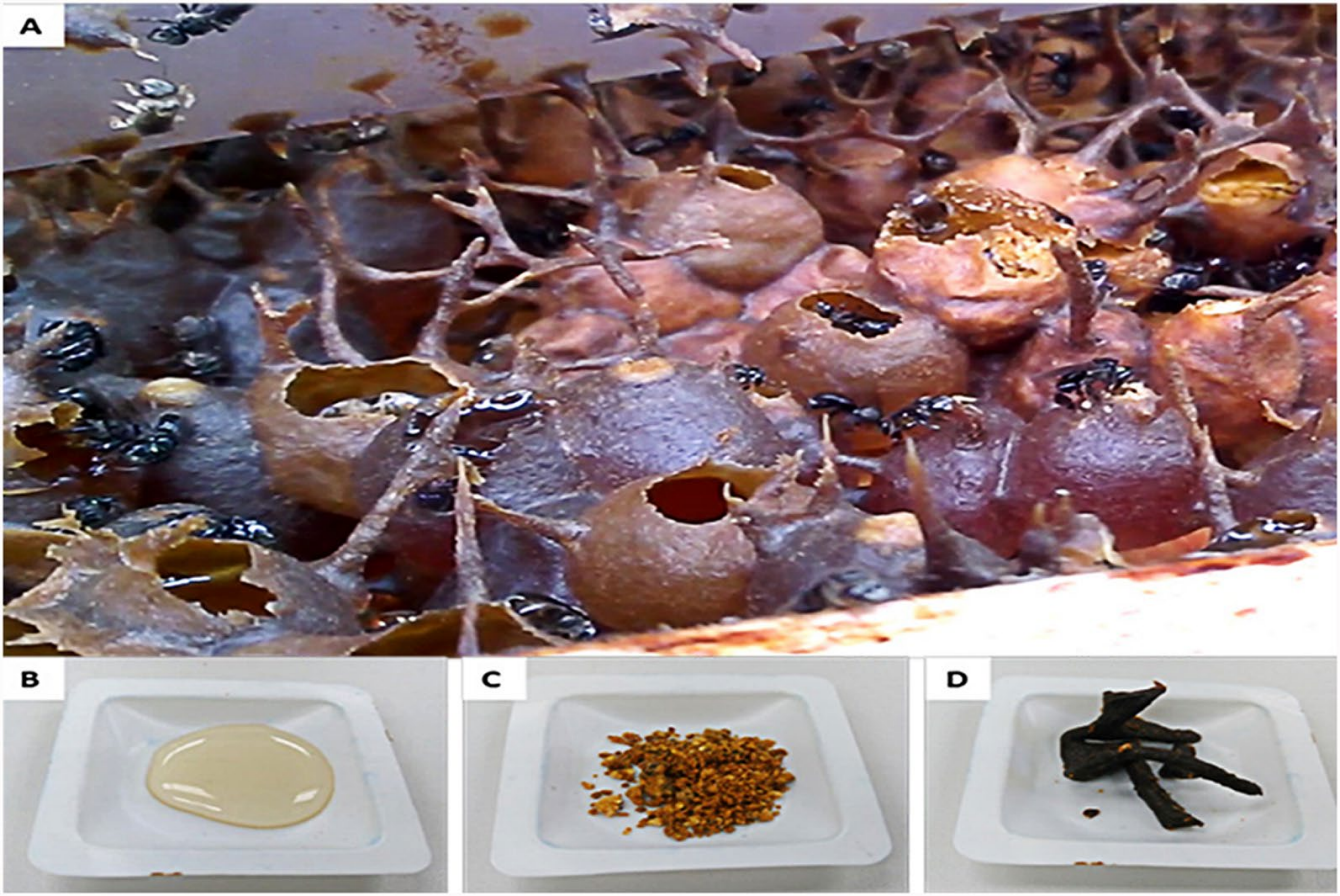
A selection of products derived from the nest of stingless bees. **a** The stingless bee nest; **b** Honey; **c** Bee bread and **d** Propolis [3]

Stingless bees have a scientific name (*Meliponini*) and belong to the eusocial insects that are formed from a monophyletic group. The monophyletic group, originally from the *Corbicolate* bees (*Hymenoptera: Apidae*) is a huge group comprising around 550 species, with 61 genes [1]. The group of crappy bees includes bumblebees (*Bombini*), honeybees (*Apini)*, and orchid bees (*Euglossini*) groups. This type of bee lives and breeds in tropical climates such as Malaysia, Australia, India, specific areas of tropical America, and parts of Africa, also in Mexico, Brazil, and Argentina. Stingless bees are not much different from other honeybees, as they live in a colony system and can produce honey, bee bread, and propolis, as shown in Fig. 1. However it lacks the property of stinging or a proper defence system, so it protects its colonies with wax-like substances that close the pores and holes [2].

Bee products, like other natural resources, are widely consumed and utilized in traditional medicine, with propolis being among the most significant. Derived from the Greek term meaning “entrance to the city,” propolis serves as a hive protector. It is a complex resinous substance partially digested by β-glycosidase in bee saliva and mixed with beeswax. Propolis exhibits temperature-dependent characteristics: it becomes soft and adhesive at higher temperatures, rigid when cooled, and transitions to liquid between 60 and 70 °C. Its color varies from green to brown and reddish, depending on its botanical origin [4].

The chemical composition of propolis varies depending on the type of plants accessible to the bees. This article reviews the active ingredients and their biological activity in propolis, based on recent studies. The gathered data may contribute to studying the safety, toxicity, and potential development of new drugs or nutritional supplements from propolis.

## Historical aspects, and traditional uses of propolis

Propolis was extensively used as a medicine by ancient civilizations such as the Greeks, Romans, and Egyptians [5]. In the Middle Ages, it lost its popularity a little, and despite this, knowledge of its medicinal properties continued in traditional folk medicine. In the Renaissance era in Europe, interest was restored in the study of natural alternatives in medicine and pharmacy, so researchers began to analyze and study the advantages of propolis, and scientists in the last century were able to prove its significance, as our forefathers had believed. Research on propolis’ chemical composition commenced during the early twentieth century and persisted beyond World War II [6].

Propolis has been utilized as a conventional remedy, since 300 BC [7] with healing activities identified by Roman and Greek doctors [8] such as Dioscorides, Galen, Aristotle, and Pliny. In 1908, the initial scientific report on propolis, which outlines its phytochemical composition and chemical properties derived from this source, has been made available to the public. Previous literature indicated that the ancient Egyptians used propolis to keep corpses from decomposing, as well as to dress and muffle wounds, and it was noted that it prevents the formation of bacteria as well as inflammation [9]. Its use as antibacterial, antifungal and antiviral medicine has expanded in many regions around the world [10]. Propolis has also been known traditionally as a local anesthetic and pain reliever in first aid for injuries [11]. In complementary medicine, propolis extracts are used to treat various medical conditions such as stomach disorders, colds and flu, and asthma in the form of sprays, powders, and ointments [12, 13]. Furthermore, propolis, both in its raw form and as extracts, is utilized in nutritional supplements, and food products targeting specific health conditions, thanks to its versatile beneficial properties [14]. The significance and advantages of propolis have gained considerable attention, leading to its inclusion in beverages and food items as a strategy to enhance immunity, fortify the body, and safeguard against illnesses [15]. Propolis has garnered interest for its potential in treating purulent disorders, promoting wound healing, aiding in burn treatment, and addressing stomach ulcers [16]. Dentistry may also benefit from propolis, as it shows promise in relieving toothaches, treating caries, and potentially impacting tooth roots [13]. In China, Propolis has been acknowledged for its medicinal properties as an anti-infection and anti-cancer agent. In England, it was recognized and adopted as a superior treatment for wounds during the seventeenth century [6]. In Indian folk medicine, propolis is widely used as a treatment for stomach ulcers [17].

Extensive historical, traditional, and scientific evidence supports propolis as a natural drug. Its medicinal properties have been acknowledged across various cultures and eras, highlighting its therapeutic value. Modern research confirms its bioeffects, reinforcing its use as a natural remedy. Its incorporation into complementary medicine and health products further reflects its growing acceptance as an effective treatment.

## Chemical analysis of propolis

Analysing the chemical composition of propolis extract is crucial for understanding its potential biological benefits and nutritional value. This information can provide valuable insights into how propolis extract may impact various health conditions.

Studies has shown that more than 420 different compounds have been characterized so far in propolis [18, 19], including countries like the Netherlands, Malaysia, Australia, New Zealand, Italy and many more. Phenolic compounds, alkenylphenol, Flavanones, flavones, flavonol, and derivatives, terpenes, and flavonoids were identified as the active ingredients responsible for the significance and efficacy of propolis [20]. Table 1 presents several compounds revealing biological activity based on chemical classification, whereas Table S1 (Supplementary Materials) shows 257 other compounds have been identified in propolis, which shows other constituents along with their respective chemical structures, emphasizing the chemical variety of propolis.

**Table 1:** Chemical components of propolis

Chemical classification	Constituents\ compounds	Structure	Bee Species /botanical Source	Bioactivity	Identification methods
Phenols	5-Pentadecyl		- *Tetrigona binghami,* - *Homotrigona fimbriata,* - *Tetrigona apicalis*	Antifungal activity [26]	NMR, LC/MS [21]
	Resorcinol		- *Tetrigona binghami,* - *Homotrigona fimbriata,* - *Tetrigona apicalis*	Antidermatophytic Action [27]	GC/EIMS [21]
	3,4-Dihydroxyphenylethanol		- Egyptian (brown), Chinese (green), and Bulgarian (brown) propolis	Anti-oxidant, and neuroprotective effect [28]	LC–MS [29]
	1,2-Dihydroxybenzene		- Egyptian (brown), Chinese (green), and Bulgarian (brown) propolis	Antimicrobial activity [30], Cytoprotective activity [31]	LC–MS [29]
Phenolic acids	Gallic acid		- *Apis mellifera*	Antioxidant [32], and antibacterial activities [33]	GC/MS [25, 34]
	Cinnamic acid		- *Apis mellifera*	Antimicrobial activity [35], Neurological Activity [36], lowering blood glucose [37], and antioxidant activity [38]	
	Caffeic acid		- *Apis mellifera*	Anti-inflammatory activity, enhancement of atherosclerosis, and Alzheimer’s disease [39]	
	3,4-Dimethyl-caffeic acid		- *Portuguese propolis*	Anticancer [40], anti-inflammatory activity [41]	LC/MS [42]
	Ferulic acid		- *Portuguese propolis*	Antioxidant [43], anti-virous [44],and anti-inflammatory activities [45]	
Flavonoids	Apigenin		- *Populus spp.*	Anticancer, Antioxidant and Anti-inflammatory Effects [46]	GC/MS [25, 47]
	Pinobanksin		- *Populus spp*	Antioxidant activity [48]	
	Kaempferol		- *Ferula spp*	Cardiovascular disease, antioxidant activity [49]	
	Pinocembrin		- *Ferula spp*	Neuroprotective against cerebral ischemic injury, anti-inflammatory, and antimicrobial activities [50]	
	Epigallocatechin		- *Ferula spp*	Anticancer activity [51]	
	Genistein		- *Ferula spp*	Antioxidant, and anticancer activities [52]	
	Pinobanksin-5-methylether acetate		- *Ferula spp*	Antimicrobial activities [53]	
	kaempferol-4’-methyl ether		- *Ferula spp*	Antimicrobial activities [54]	
	Naringenin		- *Dalbergiae castaphyllum*	Anticancer [55], and anti-inflammatory activities [56]	HPLC–DAD [57]
	Quercetin		- *Fabaceae*	Anti-Alzheimer, antioxidant activity [58], and aantibacterial activities [59]	
	Isorhamnetin		- *Fabaceae*	Antioxidant, antiviral, and antimicrobial, and antiallergic activities [60]	
	3,5,7,4′-Tetrahydroxy-3′-methoxyflavylium		- Egyptian (brown), Chinese (green), and Bulgarian (brown) propolis	Anti-inflammatory, anti-allergic, antiviral, and anticarcinogenic [61]	GC–MS [29]
Flavanones, flavones, flavonol, and derivatives	Rutin		- *Populus spp*	Antioxidant [62], antimicrobial, and anticancer activities [63]	GC/MS [25]
	Pinobanksin-3-*O*-acetate		- *Populus spp* - *Ferula spp*	Antimicrobial, and antioxidant activities [64]	
	Chrysin		- *Populus spp.* - *Ferula spp*	Hepatoprotective [65], Anticancer activities [66], and attenuate psoriasis-like skin lesions [67]	
	Gallocatechin		- *Populus spp.* - *Ferula spp*	Anti-inflammatory, anti-cancer, and antibacterial activities [68]	
	Myricetin		- *Populus spp.* - *Ferula spp*	Neuroprotective effect, antimicrobial and antioxidant activities [69]	
	Pinobanksin-3-(E)-caffeate		- *Populus spp.* - *Ferula spp*	Antimicrobial and antioxidant activities [70]	
	Catechol		- *Populus spp.* - *Ferula spp*	Wound healing [71], antioxidant and anti-inflammatory activities [72]	
	Catechin		- *Populus spp.* - *Ferula spp*	Enhancing the absorption of functional foods and their antioxidant properties [73]	
	Esculetin		- *Populus spp.* - *Ferula spp*	Antitumor [74], and antibacterial activity [75]	
	Tectochrysin		- *Populus spp* - *Ferula spp*	Anticancer [76], and decrease inflammatory markers levels [77]	
	Myricetin-3,7,3′-trimethyl ether		- *Castaphyllum*	Inhibit skin hyperpigmentation [78], treat yellow plague, malaria, and diarrhea [79]	HPLC–DAD [57]
	4,2′,4′-Trihydroxy-2-methoxychalcone		- *Fabaceae*	Antihyperuricemic and renal protective effects [80], antioxidant, and anti-inflammatory activities [81]	
	5,7-Dihydroxyflavanone		- Southern Chile propolis	Anti-inflammatory agent [82]	HPLD-DAD-ESI–MS/MS [83]
	5-Hydroxy-7-methoxyflavanone		- Southern Chile propolis	Antibacterial activity [84]	
	5,7-Dihydroxyflavone		- Southern Chile propolis	Antidiabetic properties [85]	
	3,5,7-Trihydroxyflavone		- Southern Chile propolis	Antimicrobial activity [86]	
Alkenylphenol	2-Hydroxyl-6-(14′Z-nonadecenyl) benzoic acid		- *Trigona minor*	Antimicrobial activity [87]	NMR spectroscopic analysis [88]
	3-Undecyl phenol		- Cameroonian propolis	Antinematodal activity [89]	GC/MS [90]
	3-Tetradecylphenol		- Cameroonian propolis	Potential antioxidant properties [91]	
	3-Heptadecylphenol		- Cameroonian propolis	Antiproliferative, antimicrobial, antileishmanial, antioxidant activities [92]	
	3-(12′*Z*-Heptadecenyl)-phenol		- Cameroonian propolis	Anti-inflammatory effects [93]	
Terpenoid	Germacrene D		- *Tetrigona apicalis* - *Tetrigona binghami* - *Homotrigona fimbriata*	Anti-bacterial efficacy [94]	GC–MS [94]
	Sandaracopimaric acid		- *Apis mellifera jemenitica*	Anti-inflammatory activity [95]	APCI/MS [96]
Phenylpropanoids	(E)-5-Hydroxy-1,7-diphenylhept-1-ene-3-acetate		Southern Chile propolis	Antibacterial, and antioxidant effects [83]	HPLD-DAD-ESI–MS/MS [83]
	(E)-3-Hydroxy-1,7-diphenylhept-1-ene-5-acetate				
Vitamins	Vitamin C		- *Apis mellifera caucasica*	Antioxidant activities [97]	Colorimetric method [98]
	Vitamin E		- West Amazonian Ecuador propolis - *Apis mellifera*	Antioxidant activities [99]	HPLC [100]
Amino acids	Pyroglutamic acid		- *T-apicalis*	Antifungal, and antibacterial activities [101]	Colorimetric method [98]
Aldehyde	Cyclohex-1-en-1- carboxaldehyde		- *Tetrigona apicalis* - *etrigona binghami,* - *Homotrigona fimbriata*	Antivirus effects [102]	GC–MS [98]

Medical benefits have been reported for propolis produced from Malaysian stingless bees, which are classified into 29 species Malaysian farms, and forests. The most famous species are (*Tetrigona binghami, homotrigona fimbriata* and *tetrigona apicalis*). These benefits are due to the richness of its composition of polyphenols, flavonoids, esters, terpenes, vitamins, and minerals, as well as enzymes, as it contains many phenols (5-pentadecyl and resorcinol), flavonoids (pinobanksin-5-methyl ether acetate and kaempferol- dimethyl ether), steroids (19-cyclolanost-24-en-3- ol, 9,19-cyclolanostane-3-ol, 24-methylene, and 9,19- cyclolanost-24-en-3-ol), terpenes such as (aromadendrene, *α*-eudesmol, caryophyllene oxide, and squalene), vitamins and minerals as well as enzymes [21]. In the same context, Brazilian propolis is characterized by its containing flavonoids, fatty acids, phenylpropanoids, and their derivatives, such as artibiline c, as well as chlorogenic acids [22]. Chi et al. (2020) reported that Chinese propolis contains phenolic alcohols, aldoketones, sesquiterpenoids, esters, and hydrocarbons [23]. Australian bee propolis has been shown to contain a high percentage of flavonoids (abyssinoflavanone, propolin, and nymphaeol) in addition to five-cyclic triterpenoids [24]. As for the content of bee propolis in the Mediterranean countries, it is characterized by its composition of phenols, esters, and flavonoids in its non-volatile part. The volatile part of propolis in Italy, Greece, Croatia, Egypt, Algeria and Libya was distinguished for containing benzoic acid and its esters, mono- and sesquiterpenes [25].

### Physicochemical components

**Fig. 2:**
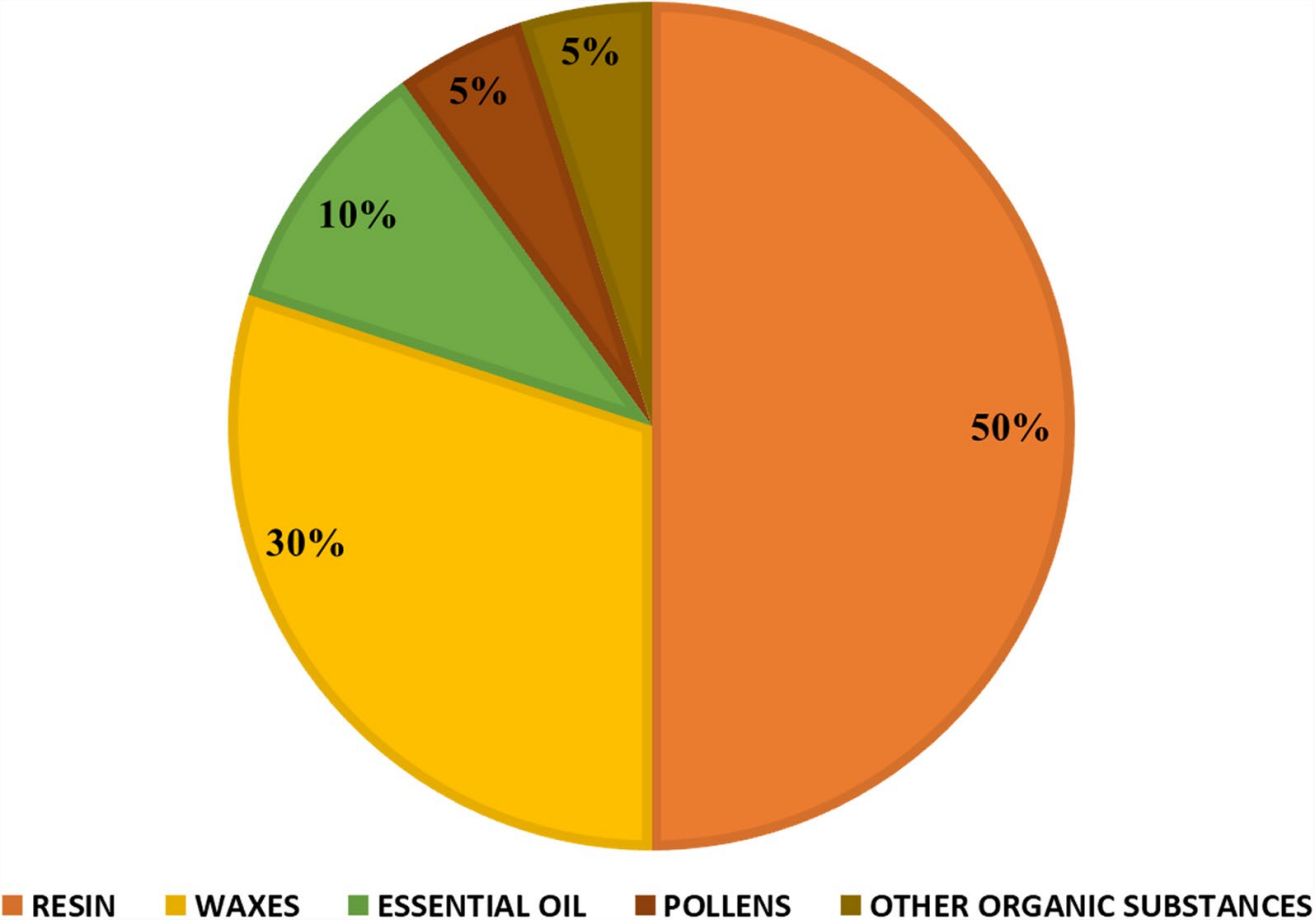
Main composition of propolis

Nature and the original botanical type play an important role in propolis’ general and exact ingredient ratios and physical characteristics. Propolis typically comprises 40–50% resins, 10% essential oils, 20- 30% wax, 5% phenols, flavonoids, and 5% pollen-containing vitamins and minerals, Fig. 2 [103]. The percentage of moisture, carbohydrates, fats, protein, fibre, ash, and wax in different types of extracted bee propolis from different plant sources in various regions around the world show in Table 2.

**Table 2:** Physicochemical components of propolis

Sample region	Bee species /Botanical source	Moisture (%)	Fat (%)	Fiber (%)	Carbohydrates (%)	Protein (%)	Ash (%)	Waxes (%)	References
Portuguese propolis (Mirandela, Mogadouro, Nogueira, and Vinhais)	- *Populus sp.,* - *Pinus sp.* - *Quercus sp.* - *Castanea* sativa	3.4–5.3	–	–	–	–	1.6–2.2	4.8–16	[107]
Malaysian propolis	- *Heterotrigona itama*	23.7	21.43	44.36	49.6	3.8	4.1	–	[47]
	- *Geniotrigona thoracica*	9.9	73.4	67.4	8.4	2.1	5.9	–	
	- *Tetrigona apicalis*	–	42.3		9.1	0.62	–		[98]
	- *Tetrigona binghami*	15.9	–	–	9.5	0.53	–		
	- *Heterotrigona fimbriata*	4.9	-	–	4.6	0.70	–		
Ethiopian propolis (Boji Dirmaji and, Fincha’a district	- *Lauraceae, Rubiaceae, Asteraceae, Euphorbiaceae*	2.22–6.89	–	–	–	–	9.6–9.7	16.7	[108]
	- *Asteraceae* - *Apocynaceae* - *Rosaceae* - *Salicaceae*	1.12–2.29	–	–	–		2.74–2.84	14.7	
Brazil (Paraná state)	- *Natura propolis*	7.4	23–25		–	28–32	2	34	[109]
Indonesia	- *Trigona sp*	7.4–8.7	38.6–61.6	45–58	25–64	2–3	1.3 –3.4	–	[105]
Northern India	- *Punjab Propolis*	4.8	53.6	3.1	–	9.4	3	22.9	[110]
	- *Rajasthan Propolis*	5.1	54.5	2.5	–	8.7	3.7	21.3	
	- *Haryana Propolis*	6.2	56.6	2.2	–	8.3	4.7	20.6	
	- *Himachal Pradesh Propolis*	7.3	68.8	1.9	–	7.2	3.5	16.5	

It is worth mentioning that the carbohydrates source in the composition of propolis are still unknown, this may be since they contain sugar alcohols and acids. The main sources of sugars (glucose, fructose, and sucrose) are nectar and honey [13].

The wax in propolis is a stable, yellow-coloured, moisture-resistant, absorbent, and non-heat-resistant substance. Wax is composed of esters, acids, alcohol, and some free hydrocarbons. In addition, other components of propolis have been reported, including amino acids, pollen, and enzymes derived from bee glandular secretions like succinic dehydrogenase, adenosine triphosphatase, glucose-6-phosphatase, acid phosphatase, α-amylase, β-amylase, α-lactamase, β-lactamase, maltase, esterase, and transhydrogenase [104].

Based on absorption spectroscopy on propolis samples, literature has shown that propolis possesses vitamins as one of the most important micronutrients directly involved in the metabolism of the human body. vitamin B complex (Thiamine, riboflavin, nicotinamide, niacin, pyridoxine, and folic acid) [105], vitamin E,,vitamin C, and vitamin A. In the same context, Propolis contains a variety of minerals which are primarily as cofactors for enzymatic activities, as (sodium, potassium, copper, zinc, iron, calcium, phosphorus, manganese, selenium, and magnesium) [106].

### Phytochemical screening

Numerous studies have shown the phytochemical makeup of propolis samples from diverse geographical regions and bee species as shown in Table 3. Portuguese propolis, sourced from *Populus sp., Pinus sp., Quercus sp.,* and *Castanea sativa*, comprises phenolic components in concentrations of 11–28% and flavonoids at 3–12% [107]. Malaysian propolis, sourced from many stingless bee species, exhibits significant compositional heterogeneity. *Tetrigona apicalis* propolis contains 7.6 mg/mL of phenolics, 34.5 mg/mL of flavonoids, and 0.66 mg/mL of terpenoids; *Tetrigona binghami* contains 10.1 mg/mL of phenolics, 34.1 mg/mL of flavonoids, and 2.1 mg/mL of terpenoids; *Heterotrigona fimbriata* has 13.2 mg/mL of phenolics, 34.5 mg/mL of flavonoids, and 1.1 mg/mL of terpenoids; whereas *Geniotrigona thoracica* propolis contains 55.1 µM GAE/g of phenolics and 326.1 µM QE/g of flavonoids [2, 98]. Chilean propolis sourced from places including Rincon de Yaquil, Cuncumen, de la Araucanía, and Metropolitana has phenolic components ranging from 11.4 to 20.8 g GAE per 100 g of methanol extract and flavonoids between 1.7 and 14 g catechin equivalents per 100 g of methanol extract [83]. In Brazil (Paraná state), natural propolis was determined to contain 4.17% phenolics and 0.26 g/100 g flavonoids [109]. Propolis from India, sourced from places including Karna, Hamirpur, Sarangpur, Palladam, and Hubli, exhibited phenolic content of 10–18 µg/g, flavonoid levels ranging from 48–98 µg/g, and terpenoid concentrations of 15–38 µg/g [111].

**Table 3:** Concentration of different phytochemical compounds in propolis

Sample region	Bee species /botanical source	Phenolic compounds	Flavonoid compounds	Terpenoid	References
Portuguese propolis	- *Populus sp.,* - *Pinus sp.* - *Quercus sp.* - *Castanea sativa*	11–28%	3–12%	–	[107]
Malaysian propolis	- *Tetrigona apicalis*	7.6 (mg/mL)	34.5 (mg/mL)	0.66 (mg/mL)	[98]
	- *Tetrigona binghami*	10.1 (mg/mL)	34.1 (mg/mL)	2.1 (mg/mL)	
	- *Heterotrigona fimbriata*	13.2 mg/mL	34.5 mg/mL	1.1 mg/mL	
	- *Geniotrigona thoracica*	55.1 µM GAE/ g of dry weight	326.1 µM QE /g of dry weight	–	[2]
Chilean propolis (Rincon de Yaquil, Cuncumen, de la Araucanía, and Metropolitana regions)	- Different types	11.4–20.8 (g GAE/100 g MeOH Extract)	1.7–14 (g Catechin Equivalents/100g MeOH Extract)	–	[83]
Brazil (Paraná state)	- Natural propolis	4.17%	0.26 (g.100g–1)	–	[109]
Indian propolis (Karna, Hamirpur, Sarangpur, Palladam, and Hubli regions)	- Different types	10–18 (µg/gm)	48–98 (µg/gm)	15–38 (µg/gm)	[111]

## Mechanism of action of propolis

Phenolic compounds, which include phenolic acids (caffeic acid, salvianolic acid B, chlorogenic acid, and ferulic acid), tannins, and stilbenes, give propolis nutritional and medicinal curative traits, such as immune system strengthening, antioxidant, anti-inflammatory, and anti-cancer properties. It is noteworthy that the presence of flavonoids in propolis assesses its quality [112]. Flavonoids such as Apigenin and Galangin display notable biological activities as mentioned in the Table 4. Apigenin exhibits potent antioxidant and anti-inflammatory properties by promoting metal chelation, neutralizing free radicals, and augmenting phase II detoxification enzymes in both cell culture and in vivo tumour models [113, 114]. It also contributes to cancer prevention by inducing apoptosis in various cell lines and animal models [115]. Galangin has shown potential in neuroprotection, particularly in Alzheimer’s and Parkinson’s disease, through the inhibition of pro-inflammatory cytokines (IL-1β, IL-6, IL-18, TNF-α) and reactive oxygen species (ROS) [116, 117]. Additionally, galangin improves insulin sensitivity by promoting glucose uptake and glycogen synthesis, increasing hexokinase (HK) and pyruvate kinase (PK) activity, and upregulating insulin receptor (IR), Akt, and GSK3β phosphorylation, while downregulating insulin receptor substrate and mTOR phosphorylation [118, 119].

**Table 4:** Mechanism of bioactive compounds in propolis

Class	Compound	Mechanism of action
Phenolic compounds	Phenolic acids (caffeic acid (CA), salvianolic acid B (SAB), chlorogenic acid (ChA) and ferulic acid)	- Antioxidant activity by Inhibits xanthine oxidase and NADPH oxidase (NADPH oxidase) [125] - Chelates metal ions (iron, copper) involved in ROS formation [126] - Inhibits cyclooxygenase (COX-1, COX-2) and lipoxygenase (5-LOX, 12-LOX, 15-LOX) [127]
	Tannic acid	- Effect on breast cancer cell lines (MCF-7) by inhibits cell proliferation differentiation and Induces apoptosis [128] - Enhance glucose uptake and inhibit adipogenesis and improve the pathological oxidative state of diabetic situation [129]
	Resveratrol and piceatannol	- Inhibit transcription factors involved in inflammation (NF-κB, AP-1, MAPKs) [130] - Reduces production of inflammatory markers (Reactive nitrogen and oxygen species, cytokines) [130] - Modulates cell proliferation and apoptosis [131] - Inhibits angiogenesis and tumor growth [132] - Protect skin against UV-induced oxidative damage [133] - Promotes wound healing and reduces inflammation [133]
Flavonoids	Apigenin	- Antioxidant and anti-inflammatory activities [113] - Facilitates metal chelation, neutralizes free radicals, and enhances phase II detoxification enzymes in cell culture and in vivo tumor models [114] - Prevention of cancer through the induction of apoptosis in several cell lines and animal models [115]
	Galangin	- Anti-neurodegenerative like, Alzheimer’s and Parkinson’s disease by inhibiting pro-inflammatory cytokines (IL-1β, IL-6, IL-18, TNF-α) and reactive oxygen species (ROS) [116, 117] - Improves insulin sensitivity by Promotes glucose uptake and glycogen synthesis Increases hexokinase (HK) and pyruvate kinase (PK) activity, upregulates insulin receptor (IR), Akt, GSK3β phosphorylation, downregulates insulin receptor substrate and mTOR phosphorylation [118, 119]
Terpenoids	*α*-Pinene	- Antifungal activity against *Candida albicans*, antibacterial against *methicillin-resistant Staphylococcus aureus* (MRSA), and inhibited phospholipase and esterase activities in *Cryptococcus* neoformans [134]
	Junipene	- Demonstrated cytotoxic efficacy against prostate (DU-145) and oral (SCC-29B) cancer cell lines [135]
	*δ-*Cadinene	- Not tested
	Isocupressic acid	- Antioxidant activity through the inhibition of mitochondrial dehydrogenase function and the suppression of superoxide generation [136] - Inhibition of methicillin resistant and *S. aureus* [136]
	Pimaric acid	- Exhibits anti-atherosclerotic activity by suppressing the production of matrix metalloproteinases and reduces the nuclear levels of NF-κB p65 and p-ATF2 proteins [137] - Inhibits the activation of tumour necrosis factor (TNF)-α-induced mitogen-activated protein kinase (MAPK) pathways, including ERK1/2, p38, and JNK [137]
	Communic acid	- Antimicrobial against Staphylococcus aureus, *S. epidermidis*, Aspergillus fumigatus, and Candida albicans [138] - Cytotoxic activity on cancer cells [139]
	14,15-Dinor-13-oxo-8(17)-labden-19-oic acid	- Antimicrobial efficacy against select Gram-positive and Gram-negative bacteria, and certain pathogenic fungi [140]
	(24(E)-Cycloart)-24-ene-26-ol-3-one	- Exhibits cytotoxicity on colon cancer cell lines via binding to tumour necrosis factor receptor 1 (TNF-R1), resulting in a decrease in mitochondrial membrane potential (MMP) and the release of cytochrome C, hence inducing apoptosis in cancer cells [141]
	20-Hydroxy-24-dammaren-3-one	- Not tested
Terpenoid derivatives	Fren-9(11)-en-2-alpha-ol	- Not tested
	Lup-20(29)-ene-3,21-dione	- Not tested
	Beta-amyrenol	- Antibacterial efficacy against *E. coli* [142]
Triterpenes	Mangiferolic acid	- Cytotoxic against gastric cancer cells [143]
	Cycloartenol	- Antidiabetic activity by lowering blood glucose, improves serum biochemical parameters, and enhances insulin release from beta cells [144]
	Ambolic acid	- Not tested

Terpenoids are approved as the most common volatile compounds in Greece propolis, among others (alpha-pinene, junipene, and δ-cadinene). Isocupressic acid, pimaric acid, communic acid, and 14,15-dinor-13-oxo-8(17)-labden-19-oic acid as the most dominant terpenoids in propolis extracts. Two diterpenes were identified from extracts of propolis in Malta and diterpene esters of hydroxybenzoic acids were isolated and bound to the plant source [120]. The extract compounds showed high antibacterial activity against *Staphylococcus aureus* [121]. Zhao et al. (2017) reported that non-stingless tropical bee propolis, such as *Heterotrigona itama* propolis in Malaysia, contains terpenoids as major biologically active compounds within 28 compounds, including (phenolic acids, flavones, terpenoids, and phytosterols), the two major terpenoids being *(*24(E)-cycloart)-24-ene-26-ol-3-one and 20-hydroxy-24-dammaren-3-one) [122]. Propolis from *Geniotrigona breastacica* bees in Kelantan, Malaysia, contained other derivatives of terpenoids, including (fren-9(11)-en-2-alpha-ol, lup-20(29)-ene-3,21-dione, and beta-amyrenol) [123]. Furthermore, Pujirahayu et al. (2019), linked the difference in *t*erpenoids to the plant source when identifying different types of triterpenes isolated from propolis extracts in Indonesia (mangiferolic acid, cycloartenol, and ambolic acid), from Mangifera indica as the plant source [124].

## Biological characteristics and therapeutic properties of propolis

**Fig. 3:**
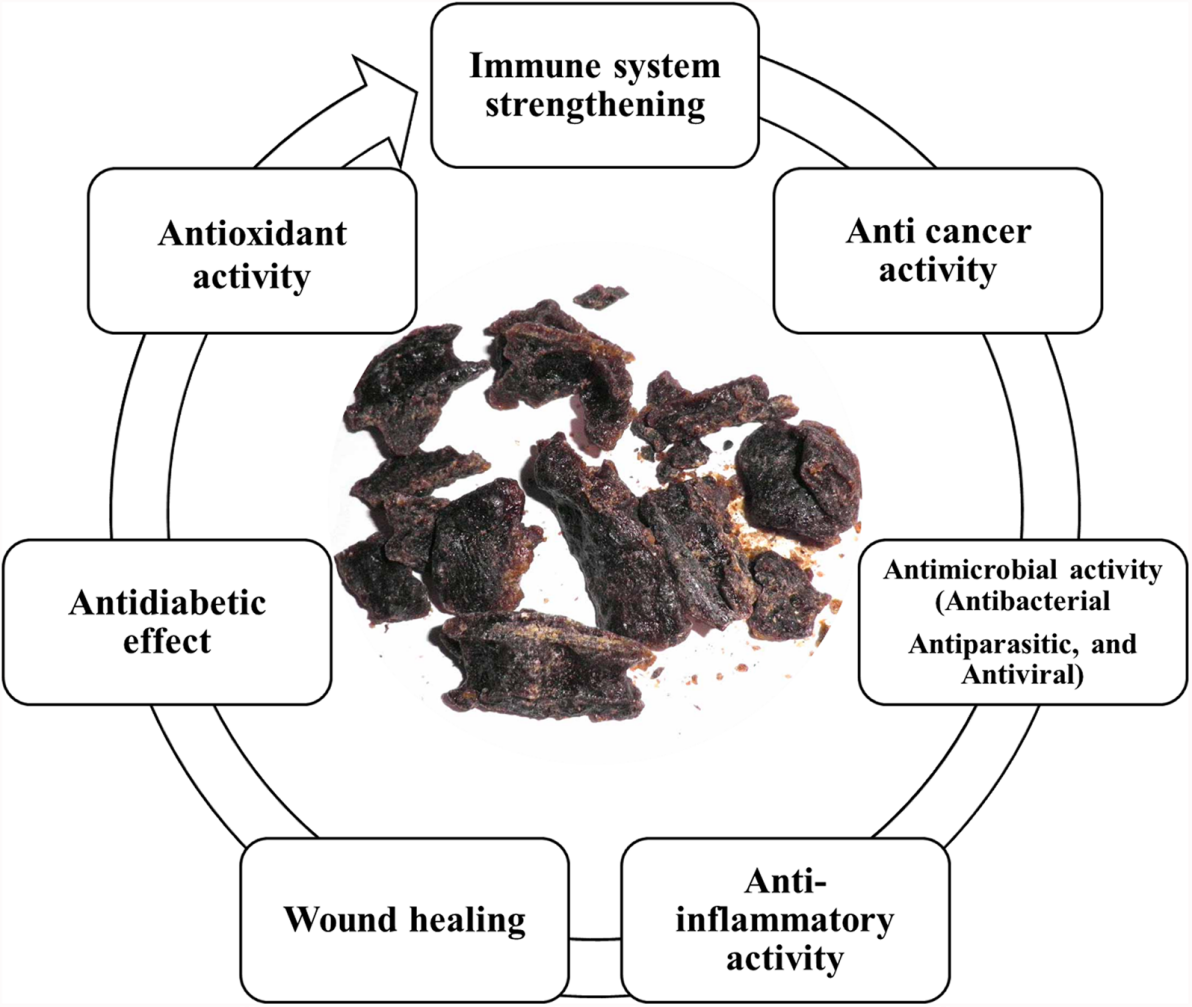
Main biological properties of propolis

The identification of the phytochemical constituents present in propolis extracts has aided in inferring the biotic and biological effects of propolis. Figure 3 shows that propolis exhibits multiple biological effects, as supported by various in vitro and in vivo studies as summarized in Table 5. The extent of its biological activity depends on factors such as the propolis chemical composition, the concentration of phytochemical compounds, as well as the extraction methods and treatments used.

**Table 5:** Summary of the biological characteristics and therapeutic properties of propolis

Source	Activity	Mechanism	References
Turkish propolis	Antibacterial activity	Effective against Gram-positive and Gram-negative bacteria (*Escherichia coli,* and *Bacillus subtilis*) by inhibition of adhesion and division, disruption of membrane potential, increased permeability of the cell membrane, reduced bacteria motility	[146]
French propolis	Synergistic effect with antibiotics on strains of methicillin-sensitive Staphylococcus aureus	Enhance effectiveness of (Ampicillin, gentamicin, and streptomycin), therefore reduce required antibiotic concentration	[147]
Brazilian nanohydroxyapatite (nanoHA) surfaces infused in propolis extracts	Biofilm inhibition	Prevents biofilm formation of *Staphylococcus aureus* biofilms	[148]
Ethanolic extracts of Turkish propolis	Antimicrobial activity	Inhibit the growth of bacterial strains, including *Streptococcus sanguinis, Streptococcus pyogenes, Streptococcus mutans*, and *C. albicans*, in a dose-dependent manner	[145]
Malaysian ethanol and n-hexane propolis extracts	Broad-spectrum antibacterial activity	Significant activity using disc diffusion and broth dilution methods on *Bacillus subtilis, Staphylococcus aureus, Escherichia coli,* and *Salmonell*a	[149]
Ethanol extracts from *Apis mellifera* and *Melipona beecheii*	Antifungal effects	A halo of inhibition was demonstrated on Candida albicans ATCC 10,231, C. glabrata, and C. krusei using an antibiotic disc diffusion plot	[150]
Ethanolic extract of Polish propolis	Antifungal activity	Significant antifungal activity against several classes of fungi, such as (C. albicans, C. glabrata, and C. krusei) by upregulation of the CDR1 gene, which encodes an efflux pump responsible for pumping out azole antifungal agents, thereby reducing their intracellular concentration and effectiveness	[151]
Argentine propolis	Antifungal activity	Isolated flavonoids (Galangin and Penocymphrene) showed fungitoxic activity by inhibiting fungal growth and hyphal extension	[153]
Brazilian red propolis	Oral pathogen inhibition	Effective against oral pathogens (Streptococcus mutans ATCC 25,175) as natural mouth rinses and toothpaste	[154]
Solvent-extracted combined propolis	Antifungal activity	Effective against Candida albicans fungi	[152]
Propolis ethanolic extract, propolis wax extract, propolis dry extract, and propolis microparticle	Antifungal activity	Fungicidal activity against *yeast*, *pseudo hyphae, hyphae* and *Candida albicans*	[156]
Brazilian green propolis extract	Biofilm inhibition in fungi	Inhibits growth and biofilm formation of *Candida albicans*	[155]
Southeastern Brazilian propolis	Immunomodulatory	Improved immune response in macrophages and mice by increased CD4 + /CD8 + T-cell ratio	[157]
General propolis extracts	Treatment of respiratory and inflammatory diseases	Enhanced immune defense and anti-inflammatory response against respiratory tract inflammation, skin ulcers, laryngotracheitis, and periodontitis	[14]
Propolis flavonoids	Vaccine adjuvant	Increased phagocytic activity and sustained antibody production enhanced lymphocyte proliferation, improved leukocyte response, reduced dosage requirement, extended vaccine efficacy, early protection, and increased non-specific immunity	[158]
Propolis combined with Taishan Pinus massoniana pollen polysaccharide	Antiviral and immunostimulatory	Reduced viral load and improved immune system response in immunosuppressive infections	[159]
Isolated propolis flavonoids (Quercetin, flavanols, and flavones)	Anti-inflammatory activity	Quercetin, flavanols, and flavones, have been demonstrated to alter inflammatory cell activity, diminish inflammatory cell infiltration and cytokine secretion, and regulate inflammatory mediators, including IL-6, TNF-α, and IL-13; they also inhibit the NF-κB, IKK, and p38 MAPK pathways	[161, 162]
Propolis supplements	Immunomodulatory	Increases CD4 + /CD8 + T-cell ratio, promotes lymphocyte proliferation, enhances leukocyte response, stimulates antibody production, strengthens immune defense and supports vaccine efficacy	[157]
Brazilian propolis isolated compounds (vestitol, artepillin C, and neovestitol)	Boosting immune response	The isolated compounds, vestitol, artepillin C, and neovestitol, influence the body’s immune response and inflammation by inhibiting inflammatory cytokines (TNF-α) and chemokines (CXCL1/KC and CXCL2/MIP2), as well as suppressing NF-κB activation. As well as they impede neutrophil adhesion and migration across barriers by affecting the expression of ICAM-1, VCAM-1, and E-selectin	[163]
Green propolis water extracts	Anti-inflammatory effects	In instances of lipopolysaccharide (LPS)-induced pneumonia, the extracts diminish macrophages and neutrophils, reduce the secretion of tumor necrosis factor alpha (TNF-α) and interleukin 6 (IL-6), while elevating the levels of interleukin 10 (IL-10) and transforming growth factor beta (TGF-β)	[164]
Red propolis	Anti-inflammatory efficacy	Reducing renal macrophage infiltration in laboratory animals (mice) with chronic kidney disease	[165]
propolis derived compounds (Caffeic acid and polyethylene)	Nitric oxide (NO) inhibition, Anti-inflammatory activity	Caffeic acid and polyethylene significantly inhibit the production of nitric oxide (NO) induced by lipopolysaccharide (LPS) in RAW264.7 macrophages. These chemicals affect transcriptional processes, demonstrating anti-inflammatory characteristics by inhibiting NF-κB and p38 MAP kinase	[166]
Brazilian propolis isolated compounds (Fesetitol and neovestitol)	Modulating inflammatory mechanisms	Fesetitol and neovestitol had effects on mice subjected to inflammation induced by lipopolysaccharide (LPS). These chemicals obstructed neutrophil transmigration by decreasing calcium influx, hence reducing the levels of CXCL1/KC and CXCL2/MIP2 and hindering neutrophil chemotaxis	[167]
General propolis exerts	Modulating inflammatory mechanisms	Propolis extracts worked in several ways, such as by blocking TLR4, MyD88, NLRP inflammasomes, NF-κB, and cytokines that cause inflammation, such as IL-1β, IL-6, and TNF-α. Additionally, it regulates CXCL9, and CXCL10, thereby limiting the migration of neutrophils and macrophages	[168]
Brazilian propolis ethanol extract	Modulate the anti-inflammatory conditions	Promoting the differentiation of M1 macrophages to D11b + , Gr-1 + myeloid-derived suppressor cells (MDSCs) in visceral adipose tissue and the peritoneal cavity of mice	[169]
Taiwanese green propolis	Anti-inflammatory and anti-gout activity	Mitigate the excessive infiltration of neutrophils induced by the injection of monosodium urate (MSU) into the abdominal cavity, and inhibit the synthesis of caspase-1, IL-1β, IL-6, and MCP-1 in the fluid utilized for peritoneal dialysis, which was stimulated by MSU	[170]
Croatian propolis extract	Improving psoriasis	Mitigating skin lipid impairment, diminishing the number of inflammatory cells in the affected skin and peritoneal cavity, and specifically inhibiting macrophage activity	[171]
Different Brazilian green propolis extracts formulations	Immune modulating	Exhibit diverse immune-modulating effects on cytokine production (IL-6, TNF-α, and IL-10) in macrophages, depending on dosage	[172]
Thai propolis extract	Anti-inflammatory material for tooth pulp capping	Inhibited COX-2 expression and PGE2 synthesis in IL-1β-treated human dental pulp cells through NF-kB activation	[173]
Propolis bioactive compounds	Vaccine applications	The active components in propolis promote the synthesis of substantial quantities of antibodies and facilitate lymphocyte proliferation, hence augmenting the potential of propolis in vaccine applications	[174]
Brazilian propolis	Anti-atherosclerotic activity	Improve the plasma lipid profile, stabilize atherosclerotic plaques, and reduce macrophage apoptosis, vascular smooth muscle proliferation, and metalloproteinase activity	[175]
Chinese propolis extracts	Vascular anti-inflammatory activity	Reduce blood pressure and enhance cardiac function in hypertensive rats via processes that include the suppression of catecholamine production and endothelium-dependent vasodilation	[176]
Croatian propolis extract	Lipid profile improvement	Propolis extract administration for 30 days in male rats reduced LDL levels and increased HDL levels, improving factors linked to atherosclerosis	[177, 178]
General propolis extract	Anti-atherosclerotic and anti-dyslipidemia effect	Decreases LDL oxidation in mice by activating Nrf2 and enhancing antioxidant enzymes, suppresses inflammation-associated enzymes ADAM10 and ADAM17 in mice, and significantly reduces atherosclerosis and dyslipidemia	[179]
Malaysian propolis extract	Lipid profile improvement	Diminishes foam cell production in macrophages by restricting ox-LDL absorption and cholesterol ester buildup, thereby reducing pro-inflammatory cytokines, facilitating lipid breakdown, and decreasing total cholesterol associated with atherosclerosis	[180]
Malaysian stingless bee *Geniotrigona thoracica* propolis	Cardioprotective and antioxidant activity	Inhibits LDL oxidation by activating Nrf2, enhancing antioxidant enzyme activity, and supporting phase II detoxification and GSH metabolism	[181]
Caffeic acid phenethyl ester isolated from Polish propolis	Cardioprotective and antioxidant activity	Protects the cardiovascular system by modulating cytokines in a dose-dependent manner	[182]
General propolis extracts	Antioxidant and cardioprotective activity	Neutralized free radicals and safeguarded red blood cells from oxidative damage, diminishing lipid peroxidation indicators	[146]
propolis drops	Enhancement of redox balance	Administration of 15 propolis drops twice daily for 90 days resulted in a 67% reduction in lipid peroxidation markers, a 175% increase in glutathione levels, and improved HDL levels compared to the placebo group	[183]
Propolis supplements	Improved antioxidant status in diabetes	Propolis supplementation increased glutathione (GSH) levels, diminished protein oxidation, and decreased lactate dehydrogenase activity. It reduced blood TNF-α levels, elevated IL-1β and IL-6 levels, but did not influence glucose, HbA1c, insulin, aldose reductase, or adiponectin levels	[184]
Polyphenolic propolis compounds	Antioxidant activity	Polyphenolic derivatives showing higher antioxidant capacity in ABTS and DPPH assays	[185]
Malaysian propolis	Cardioprotective potential	The treatment of Malaysian propolis (100 mg/kg/day for 30 days) significantly decreased cardiac enzyme markers, troponin I, and lipid peroxides, while simultaneously enhancing antioxidant defense enzymes in a rat model of isoproterenol-induced myocardial infarction	[186]
Malaysian stingless bee species (*Tetrigona apicalis, Heterotrigona itama*, and *Geniotrigona thoracica*)	Polyphenol-linked antioxidant activity	Significant antioxidant activity in vitro assays, positively correlated with polyphenol concentration	[2]
Brazilian propolis	Anticancer and antioxidant efficacy	Inhibits angiogenesis, reduces cell proliferation, promotes apoptosis, reduces oxidative stress, and enhances antioxidant enzyme activity	[188]
Propolis nutritional supplement	Chemotherapy and radiotherapy cases	Alleviates side effects during chemotherapy and radiotherapy	[189]
Brazilian propolis (*Plebeia droryana* and *Apis mellifera*)	Reduce oxidative stress	Significant antioxidant and cytotoxic effects; suppressed lipid peroxidation and protected erythrocytes from oxidative hemolysis	[190]
Brazilian propolis (*bipunctata* and *anthidioides*)	Anti-cancer efficacy	Notable cytotoxicity on human melanoma cells (SK-MEL-28)	[191]
Malaysian propolis (*Tetrigona apicalis*)	Anti-cancer efficacy	Induces apoptosis of breast cancer cells (MCF7) due to phenolic chemicals	[192]
*Trigona Sirindhorn* propolis extract	Anti-cancer efficacy	Reduce viability of head and neck cancer cells (HN30)	[193]
*Tetragonula pagdeni* propolis extract	Anti-cancer efficacy	Strong anticancer activity on human cancer cell lines (KB, HepG2, CacoEL-2, SK) with low toxicity to normal cells; attributed to gamma and alpha mangosteen components	[194]
*Trigona laeviceps* propolis ethanolic extract	Cytotoxicity effects	Marked cytotoxicity while maintaining normal cell integrity ChaGo, KATO-III, SW620, and HepG2 carcinoma cell lines	[195]
*Populus nigra* propolis extract	Anti-cancer efficacy	Concentration-dependent reduction in cancer cell volume	[196]
General propolis extract	Cytotoxicity effects	Cytotoxic effects, cell cycle arrest, apoptosis, and autophagy through the modulation of β-catenin, p53, NF-κB, MAPK, and ERK1 pathways	[197]
Isolated propolis compounds (Chrysin and kaempferol)	Stimulation of skin cell reproduction and tissue growth	Decrease cytokine synthesis in mast cells, enhance fibroblast proliferation, facilitate tissue remodelling, and accelerated recovery of burned tissues	[198]
General propolis extract	Accumulation of glycosaminoglycans in wound area	Propolis increased glycosaminoglycan levels, which are crucial for granulation, tissue growth, and wound closure	[199]
Topical propolis cream	Healing of diabetic foot ulcers and chronic wound infections	Topical administration of propolis diminishes inflammation and accelerates wound healing. Alongside substantial wound healing in diabetic foot ulcers and persistent wound infections	[200]
Propolis formulation	Antibacterial efficacy and wound healing	Propolis formulation (3.6%) shows antibacterial activity against *P. aeruginosa, K. pneumoniae, E. coli, S. aureus*, and *S. epidermidis*. Improved wound healing and reduced infection	[201]
Olyurethane composite porous foam infused with nano lignin and coated with green propolis	Enhanced mechanical properties and antibacterial activity	Improved mechanical stability, chemical resistance, antibacterial activity, and accelerated wound healing in live models	[202]
Self-emulsifying formulation with propolis	Facilitating wound healing	The self-emulsifying formulation incorporating propolis into bacterial cellulose membranes demonstrated significant wound healing efficacy, with complete healing observed within one week	[203]
Propolis isolated Caffeoylquinic acid	Anti-diabetes	Inhibits β-glucosidase and α-amylase, reducing glucose levels	[204]
General propolis extract	Antihyperglycemic effect	Reduced glucose levels and lipid peroxidation in diabetic rats in a study compared to Nigella sativa	[205]
Zinc oxide nanoparticles and propolis combination	Enzymes inhibition and oxidative stress reduction	Zinc oxide nanoparticles in conjunction with propolis (Pro-ZnO NPs) neutralized free radicals and reduced α-amylase and α-glucosidase activities	[206]
Different propolis extracts	α-Glucosidase inhibition	Glucose absorption was reduced through the inhibition of α-glucosidase activity by propolis extracts, with genistein, apigenin, kaempferol, chrysin, and luteolin acting as key active components	[207]
Propolis flavonoids	Insulin resistance reduction	Flavonoids like naringenin replicate insulin effects and reduce resistance	[207]
Iranian propolis	Glucose and HbA1c reduction	Inhibits α-glucosidase activity, lowering blood sugar levels and stimulating pancreatic beta cell activation, thereby improving carbohydrate metabolism	[208]
Propolis supplementation	Reduce blood glucose	Propolis administration over a two-month period reduced fasting blood glucose levels and glycosylation in persons with diabetes	[209]

### Antimicrobial activity

Given the urgent need to discover new natural remedies against infectious diseases, especially since some pathogens can be resistant to repeated antibiotics, propolis has been shown to be effective against Gram-positive and Gram-negative bacterial strains, as well as anaerobic and aerobic bacteria. The activity of propolis on bacterial strains occurs in two ways: firstly, by direct action on bacteria, and secondly, by stimulating the immune system (the immune system of host cells) and thus activating natural defence mechanisms. Possible mechanisms of action include inhibition of bacterial adhesion and division, reduction of their motility, disruption of membrane potential, and increased permeability of the cell membrane [145].

Studies indicate that propolis exhibits stronger activity against Gram-positive bacteria compared to Gram-negative bacteria. Its effects on bacterial strains such as (*Escherichia coli,* and *Bacillus subtilis*) include altering membrane permeability and disrupting potential, ultimately inhibiting bacterial movement [146]. A phenolic extract of propolis has been suggested as a supportive agent for antibiotics in treating Gram-positive bacterial infections. Propolis enhanced the effectiveness of ampicillin, gentamicin, and streptomycin, reducing the required antibiotic concentrations. A study on French propolis demonstrated varying effects of ethanolic extracts on 12 strains of *methicillin-sensitive Staphylococcus aureus* (*MSSA*) when combined with 10 anti-staphylococcal drugs [147]. Propolis showed the same results on *S. aureus*. As well as, it is proven the efficacy of Brazilian nanohydroxyapatite (nanoHA) surfaces infused in propolis extracts in preventing the growth and formation of biofilms of S. aureus bacteria, and staphylococcal biofilm formation reported [148].

In a comparative study between 48 ethanolic extracts of Turkish propolis against bacterial strains (*Streptococcus sanguinis, Strep- tococcus pyogenes, Streptococcus mutans,* and *C. albicans*) in addition to chemical analysis and examination of other biological activities. It was observed that the highest antimicrobial activity was due to samples with a higher content of flavonoid, phenols, cinnamic, ferulic, caffeic, chlorogenic and coumaric acids [145]. In evaluating the effect of Malaysian ethanol and n-hexane propolis extracts from the plant source *Heterotrigona itama*, Disc diffusion and broth dilution methods were used; the extracts showed significant activity against all studied bacterial strains (*B. subtilis, S. aureus, E. coli* and *Salmonella*) [149]. Ramón-Sierra et al. (2019) reported significant antifungal activity (against *C. albicans ATCC 10,231*) of ethanol extracts of propolis originating from two species of stingless bees, *Apis mellifera* and *Melipona beecheii* [150]. The ethanolic extract of Polish propolis manifest antifungal activity against several classes of fungi, such as (*C. albicans, C. glabrata,* and *C. krusei*) [151]. Moreover, a French study indicated the effectiveness of propolis extract dissolved in methanol and dichloromethane that are active against *C. albicans* and *C. glabrata* [152]. The antifungal activity of Argentine propolis has been attributed to the isolated compounds Galangin and Penocymphrene [153]. Inhibitory activity of Brazilian red propolis has been reported for oral pathogens as well as for all oral pathogens (*S. mutans ATCC 25,175, S).* The results of this study were taken into consideration as a preparation for applications of natural alternatives to oral rinses and toothpaste [154].

Moreover, research has demonstrated that Brazilian green propolis extract can effectively hinder the growth and formation of biofilms by Candida albicans, a fungus responsible for vulvovaginal candidiasis [155]. On other hand, propolis has fungicidal properties, including propolis ethanolic extract, propolis wax extract, propolis dry extract, and propolis microparticle, against three different morphotypes of *C. albicans* (yeast, pseudo hyphae, and hyphae) [156].

### Immune system strengthening

In laboratory tests, propolis demonstrated its immunomodulatory properties on macrophages, while in live mice, it increased the ratio of CD4^+^/CD8^+^ T-cells [157]. The results shed light on why propolis is used to treat different ailments like respiratory tract inflammations (both acute and chronic), skin ulcers, pharyngotracheitis, and periodontitis [14]. Studies have shown that propolis, used as a vaccine adjuvant, enhances immune defense by boosting phagocytic activity and promoting sustained antibody production and mucosal immunity. It stimulates lymphocyte proliferation, augments leukocyte response, reduces required dosage, extends vaccine efficacy, initiates early protection, and improves non-specific immunity [158]. According to B Li et al. (2015), the combination of propolis and Taishan Pinus massoniana pollen polysaccharide had a beneficial impact on the immune system and reduced the viral load in study samples suffering from immunosuppressive viral infections. This result emphasises the potential of employing natural remedies to enhance prevention and treatment strategies for immunosuppressive diseases [159].

### Anti-inflammatory capacity

Inflammation is a complex process involving chemical signals that initiate and sustain healing after tissue damage, progressing through acute and chronic phases. During the acute phase, immune cells are activated and migrate to the affected site, releasing growth factors, reactive oxygen/nitrogen species, and cytokines (Pahwa, Goyal et al., 2021). Uncontrolled acute inflammation can transition into chronic inflammation, contributing to the development of various diseases [160].

Propolis has a significant anti-inflammatory activity due to its role in regulating inflammatory mediators. Several chemical compounds isolated from propolis have proven anti-inflammatory efficacy, where the biological activity of flavonoids, including quercetin, flavanols and flavones has been shown to modulate inflammatory cell function [161]. Chrysin, cearoin, (4-methoxydalbergion), and (3′,4′-dihydroxy-4-methoxydalbergione) inhibit expression of inflammatory gene expression in bone marrow-derived cells (*BMMC*) such as (IL-6, TNF-α, and IL-13), in addition to inhibiting IKK stimulation, result in IκBα degradation and inactivation of nuclear factor-κB (NF-κB) [162]. These results are in agreement with Franchin et al. (2018); the biological components isolated from Brazilian propolis, vestitol, artepillin C, neovestitol significantly affect the body’s immunity and its response to inflammation by inhibiting inflammatory cytokines (TNF-α) and chemokines (CXCL1/KC and CXCL2/MIP2), inhibition of NF-κB; in addition to suppressing neutrophil attachment and movement across barriers (ICAM-1, VCAM-1 and E-selectin expression) [163]. In a similar vein, Green propolis water extracts were found to have anti-inflammatory effects in LPS-induced pulmonary inflammation. They decreased macrophages and neutrophils and reduced TNF-α and IL-6 secretion, while increasing IL-10, and TGF-β levels [164]. Another study on red propolis reported its significant efficacy in reducing renal macrophage infiltration in laboratory animals (mice) with chronic kidney disease [165]. The presence of caffeic acid and polyethylene compounds derived from propolis effectively hinders the generation of nitric oxide (NO) caused by lipopolysaccharide (LPS) when acted upon by RAW264.7 macrophages. These compounds exert their influence on the transcriptional level, thereby exhibiting anti-inflammatory properties through the suppression of NF-κB and p38 MAP kinase [166]. Fesetitol and neovestitol compounds from Brazilian propolis exhibited comparable effects in a study using laboratory mice to induce inflammation with lipopolysaccharide (LPS). These compounds hindered neutrophil transmigration by blocking calcium influx, consequently inhibiting the levels of CXCL1/KC and CXCL2/MIP2 and impeding neutrophil chemotaxis [167].

Propolis exerts its effectiveness by modulating inflammatory mechanisms and transforming the environment into an anti-inflammatory state. Its anti-inflammatory effects involve multiple mechanisms, including the inhibition of TLR4, MyD88, NLRP inflammasomes, NF-κB, as well as pro-inflammatory cytokines like IL-1β, IL-6, and TNF-α. Additionally, propolis regulates CXCL9 and CXCL10, limiting the migration of neutrophils and macrophages [168]. A study showed propolis to modulate the anti-inflammatory conditions by promoting the differentiation of M1 macrophages to D11b + , Gr-1 + myeloid-derived suppressor cells (MDSCs) in visceral adipose tissue and the peritoneal cavity of mice [169]. Moreover, Hsieh et al. (2019) reported that propolis reduced the excessive entry of neutrophils caused by injecting monosodium urate (MSU) into the abdominal cavity. Additionally, propolis suppressed the production of caspase-1, IL-1β IL-6, and MCP-1 in the fluid used for peritoneal dialysis, which was induced by MSU [170].

Croatian propolis extract shows potential in improving psoriasis that are caused by irritant substances. It achieves this by reducing skin lipid damage, decreasing the presence of inflammatory cells in the injured skin and peritoneal cavity, and specifically suppressing the performance of macrophages [171]. Zamarrenho et al. (2023) investigated the effectivness of different propolis extracts formulations on cytokine production in macrophages, focusing on IL-6, TNF-α, and IL-10. The extracts showed varied immune-modulating effects [172]. Furthermore, the application of Thai propolis extract in non-toxic amounts inhibited COX-2 expression and PGE2 synthesis in IL-1β-treated human dental pulp cells through NF-kB activation. This anti-inflammatory effect highlights its potential as a therapeutic material for pulp capping [173]. The potent elements present in propolis encourage the generation of a high quantity of antibodies and promote the growth of lymphocytes, which is associated with its ability to minimize inflammation. This connection strengthens the potential application of propolis in vaccines [174].

On other hand, Propolis, with its rich chemical composition, has demonstrated significant effects on atherosclerosis. Studies indicate that propolis extracts enhance the plasma lipid profile, stabilize atherosclerotic plaques, and mitigate macrophage cell death, vascular smooth muscle proliferation, and metalloproteinase activity [175]. Additionally, Chinese propolis extracts have been shown to lower blood pressure and improve myocardial function in hypertensive rats through mechanisms involving catecholamine synthesis inhibition, endothelium-dependent vasodilation, and vascular anti-inflammatory activity [176].

A study involving male rats shown that 30 days of propolis extract administration decreased LDL levels and elevated HDL levels, hence alleviating variables associated with atherosclerosis [177]. Mice treated with propolis demonstrated reduced LDL levels compared to untreated mice, underscoring the significance of oxidized LDL (Ox-LDL) in atherosclerosis [178]. Yigit et al. (2024) found that propolis diminishes LDL oxidation in mice by activating Nrf2 and augmenting antioxidant enzymes. Moreover, propolis suppressed inflammation-associated enzymes ADAM10 and ADAM17 in mice, markedly diminishing atherosclerosis and dyslipidaemia [179].

Propolis reduces foam cell formation in macrophages by limiting ox-LDL uptake, and cholesterol ester accumulation, thereby lowering pro-inflammatory cytokines and promoting lipid degradation. Malaysian propolis extract further inhibits lipid buildup in ox-LDL-treated macrophages, reducing total cholesterol and cytokines linked to atherosclerosis [180]. It additionally inhibits LDL oxidation by activating Nrf2 and augmenting the activity of antioxidant enzymes, encompassing phase II detoxification and GSH metabolism [181]. Furthermore, caffeic acid phenethyl ester (CAPE) from Polish propolis protects the cardiovascular system by modulating cytokines in a dose-dependent manner [182].

### Antioxidant activity

Oxidative stress assumes an important function in the development of numerous human ailments, including neurodegenerative disorders, cardiovascular conditions, cancer, and diabetes [178]**.**

Propolis extracts efficiently neutralized free radicals and safeguarded red blood cells from oxidative injury, as evidenced by reduced a biomarkers of lipid peroxidation [146]. Participants taking 15 propolis drops twice daily for 90 days showed a 67% decrease in lipid peroxidation markers, a 175% increase in glutathione levels, and improved HDL levels compared to the placebo group. These findings suggest propolis enhances redox balance and may reduce cardiovascular risk [183]. Another study investigated the effects of propolis supplementation (900 mg/day/8 weeks) on antioxidant status in individuals with type 2 diabetes. Propolis increased glutathione (GSH), decreased markers of protein oxidation, and decreased lactate dehydrogenase activity. Serum TNF-α levels decreased, while IL-1β and IL-6 levels increased, but no changes were observed in glucose, HbA1c, insulin, aldose reductase, or adiponectin levels [184].

The relationship between polyphenolic derivatives and antioxidant activity using water and ethanolic solutions. UHPLC-MS identified 21 polyphenolic derivatives, with percentages varying by solvent. Ethanolic extracts showed a broader range of polyphenolics and higher antioxidant capacity in ABTS and DPPH assays [185]. Furthermore, the cardioprotective potential of Malaysian propolis was demonstrated using DPPH and FRAP assays and an isoproterenol-induced myocardial infarction rat model. Administering 100 mg/kg/day of propolis for 30 days significantly reduced cardiac enzyme markers, troponin I, and lipid peroxides, while enhancing antioxidant defence enzymes [186]. Likewise, the antioxidant properties of propolis are linked to compounds like flavonoids, phenolic acids, terpenoids, where propolis from three Malaysian stingless bee species (*Tetrigona apicalis, Heterotrigona itama*, and *Geniotrigona thoracica*) showed significant antioxidant activity in vitro assays. A positive correlation was found between polyphenol concentration and antioxidant efficiency [2].

### Anti-cancer activity

Cancer is described as a complex disease in which there is uncontrolled growth of abnormal cells, and it’s therapies still have several limitations, such as the occurrence of side effects and unintended effects on areas of the body that were not the intended target [187].

Bioactive compounds in propolis exhibit indirect anti-cancer effects by inhibiting angiogenesis, reducing cell proliferation, and promoting apoptosis, thereby affecting disease progression [188]. It modulates the tumour microenvironment, combats drug resistance, and acts as a chemo preventive agent by reducing tumour growth and oxidative stress while enhancing antioxidant enzyme activity. Additionally, propolis serves as a nutritional supplement to alleviate side effects during chemotherapy and radiotherapy [189]. Bonamigo et al. (2017) studied Brazilian propolis extracts from *Plebeia droryana* and *Apis mellifera*, finding significant antioxidant and cytotoxic effects, by suppressed lipid peroxidation caused by *AAPH,* protecting erythrocytes from oxidative haemolysis and reducing *MDA* levels [190]. Two Brazilian propolis species, *bipunctata* and *anthidioides*, exhibited notable cytotoxicity against human melanoma cells (SK-MEL-28) [191]. The propolis from Malaysian (*Tetrigona apicalis*) demonstrated apoptosis-inducing effects in *MCF7* breast cancer cells, ascribed to phenolic chemicals [192]. Moreover Trigona Sirindhorn propolis diminished the viability of head and neck cancer cells (*HN30*) in viability compared to the control [193].

The effects of *Tetragonula pagdeni* propolis were evaluated on human cancer cell lines (*KB, HepG2, CacoEL-2, SK*), revealed strong anticancer activity with low damage to normal cells, possibly attributable to gamma and alpha mangosteen components [194]. Likewise, the ethanolic extract of *Trigona laeviceps* shown significant cytotoxicity towards ChaGo, KATO-III, SW620, and HepG2 cancer cell lines while preserving the integrity of normal cells [195]. The anticancer capabilities of *Populus nigra* propolis demonstrated a concentration-dependent decrease in cancer cell volume, endorsing its application in functional food supplements for pharmacological advantages [196]. Furthermore, Altabbal et al. (2023) established that propolis elicits cytotoxic effects on cancer cells, halts the cell cycle, and initiates apoptosis and autophagy by modulating signalling pathways including β-catenin, p53, NF-κB, MAPK, and ERK1, thereby impeding tumour advancement [197].

### Wound healing properties

Propolis, has emerged as a promising option for burn management due to its skin-friendly nature, minimal risk of allergic reactions, and lack of toxicity. Additionally, its ability to stimulate skin cell reproduction, activation, and growth contributes to various biological effects that expedite the healing process, which makes it an excellent choice for this purpose.

Chrysin and kaempferol, flavonoids found in propolis, diminish cytokine production in mast cells, the remodeling phase, macrophages recruited by mast cells stimulate fibroblast proliferation and tissue remodeling [198]. According to Olczyk et al. (2013), propolis has been shown to accelerate the healing of burnt tissues by promoting the accumulation of glycosaminoglycans in the wound area, which are important in the processes of granulation, tissue growth, and wound closure [199]. The topical application of propolis is an efficacious approach for managing diabetic foot ulcers, resulting in substantial wound healing in a brief timeframe, and is effective in mitigating skin infections in individuals with chronic wound infections [200]. Additionally, the propolis formulation (3.6%) was determined is optimum for wound healing in Wistar rats, exhibiting antibacterial efficacy against pathogens such as *P. aeruginosa, K. pneumoniae, E. coli, S. aureus, and S. epidermidis* [201].

A polyurethane composite porous foam created and infused with nano lignin and coated with green propolis for use as a wound dressing. The propolis coating improved mechanical characteristics, chemical stability, and antibacterial efficacy against Staphylococcus aureus and Escherichia coli, while facilitating wound healing in live animal experiments [202]. In the context, Marquele-Oliveira et al. (2019) developed bacterial cellulose membranes including propolis through a self-emulsifying formulation, exhibiting facilitating wound healing within one week [203].

### Anti-diabetes effect of propolis

Diabetes is a metabolic disorder characterized by insufficient insulin production, leading to elevated blood glucose levels. Propolis extract show antihyperglycemic properties due to its caffeoylquinic acid (CQA) concentration, which inhibits both beta-glucosidase and alpha-amylase [204]. Clinical study demonstrated that propolis extracts reduced glucose levels and protected against lipid peroxidation in diabetic rats, compared to Nigella sativa [205].

Zinc oxide nanoparticles integrated with propolis (Pro-ZnO NPs) demonstrate promise in diabetes management by neutralizing free radicals and inhibiting the enzymes α-amylase and α-glucosidase [206]. Farida et al. (2023) emphasized the effectiveness of different propolis extracts in inhibiting α-glucosidase. Key components such as genistein, apigenin, kaempferol, chrysin, and luteolin are recognized as active constituents in propolis for diabetes, whilst flavones like naringenin replicate insulin actions and diminish resistance [207]. Iranian propolis reduces blood sugar, insulin, and glycosylated haemoglobin (HbA1c) levels by blocking α-glucosidase activity during carbohydrate metabolism and enhancing insulin secretion through the activation of pancreatic beta cells [208]. Furthermore, the consumption of propolis supplements by individuals with diabetes for a period of two months resulted in a significant decrease in both fasting blood glucose levels and glycosylation [209].

## Applications of propolis as a functional food

**Fig. 4:**
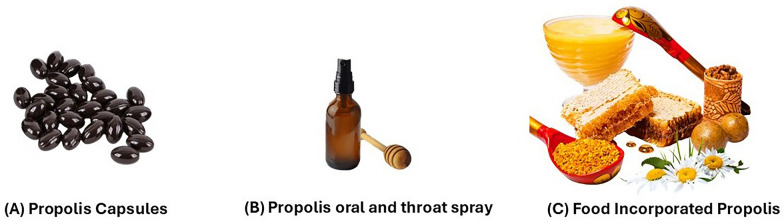
Propolis applications

Consumers shift toward natural products, the demand for functional foods has risen, positioning propolis as a key ingredient due to its biological and therapeutic properties. Propolis supplements, available globally in capsules, sprays, powders, and cosmetics (Fig. 4), have shown efficacy in managing chronic conditions like metabolic syndrome [210]. Furthermore, Yazgan et al. (2020 demonstrated the effectiveness of water- and alcohol-based propolis extracts in extending the shelf life of vacuum-packed sardines [211], while Pobiega et al. (2019) highlighted its ability to reduce lipid oxidation in meat products [212]. In dairy, red propolis extract enhanced yogurt preservation as an alternative to potassium sorbate [213]. Alvarez et al. (2017) reported that combining propolis extract with heat and ultrasound preserved the quality of fresh-cut vegetables [214].

Propolis poses challenges for food applications due to its strong sensory properties such as smell, colour, and taste. Ray et al. (2016) proposed encapsulating its active components to mask flavour and protect antioxidants, using nanoparticles formed by enclosing the active agent within a wall substance [215]. Spray drying, enhanced by additives like maltodextrin and Arabic gum, has proven effective for improving properties of encapsulated propolis. As well as propolis-based protective layers on fruit surfaces improve texture preservation, reduce industrial packaging needs, and extend shelf life while preventing microbial contamination [216].

## Discussion

Propolis has garnered significant attention due to its diverse phytochemical constituents and extensive biological activities, supported by numerous in vitro and in vivo studies. Rich in bioactive compounds such as flavonoids, phenolic acids, and terpenes, propolis demonstrates antioxidant, anti-inflammatory, antibacterial, anticancer, antidiabetic, and immunomodulatory properties, making it highly valuable for disease prevention and therapeutic support.

Comparative analyses of Brazilian, Chinese, Malaysian, and Australian propolis have shown that variations in botanical sources, environmental conditions, and bee species significantly influence chemical composition and biological efficacy. Brazilian green propolis, characterized by high levels of artepillin C and caffeic acid phenethyl ester (CAPE), exhibits strong anti-inflammatory and antioxidant properties. Malaysian propolis is abundant in flavonoids and terpenes, enhancing its antimicrobial and wound-healing capacities. Chinese propolis contains unique phenolic alcohols and sesquiterpenes, providing notable cardioprotective and anti-inflammatory effects, while Australian propolis, rich in flavonoids and triterpenoids, possesses significant antimicrobial and anticancer potential.

Propolis effectively modulates inflammatory pathways, including NF-κB, reducing cytokines such as TNF-α and IL-6, thus demonstrating efficacy in managing chronic inflammatory conditions like atherosclerosis and rheumatoid arthritis. Additionally, its immunomodulatory properties—such as promoting macrophage differentiation and enhancing antibody production—highlight its promising application in vaccine development and immune therapy. Its antioxidant activity, attributed mainly to flavonoids and phenolic acids, neutralizes reactive oxygen species, reducing oxidative stress and protecting against cardiovascular diseases and cancer. The antibacterial properties of propolis further extend their therapeutic relevance, especially against antibiotic-resistant pathogens, indicating potential as an alternative or adjunctive treatment.

Despite these promising findings, critical issues remain. Variability in chemical composition among different propolis types necessitates standardized extraction and formulation protocols to ensure consistency and reproducibility. Additionally, the bioavailability and pharmacokinetics of propolis are challenging due to its poor solubility and rapid metabolism. Advanced delivery systems, including nanotechnology-based encapsulation, have been proposed, though clinical validation is still required. Further research into potential allergic reactions and long-term toxicity is also essential for ensuring safety.

Moreover, the dual effects of propolis, acting as an antioxidant at low concentrations and a pro-oxidant at high concentrations, complicate therapeutic applications and require precise dose determination. The promising anticancer potential of propolis, involving apoptosis induction and modulation of the tumor microenvironment, demands comprehensive evaluation in controlled clinical settings due to variability in response among different cancers and possible interactions with conventional treatments.

Clinical trials have yielded mixed outcomes, emphasizing the necessity for large-scale, rigorously designed studies to conclusively establish efficacy, optimal dosing, and safety propolis. Establishing regulatory frameworks and standardized guidelines will further enhance the credibility, safety, and widespread acceptance of propolis-based therapeutic products in modern medical practice.

## The main research findings

Propolis demonstrates various biological actions, encompassing antioxidants, anti-inflammatory, antibacterial, anticancer, antidiabetic, and immunomodulatory properties. Green Brazilian propolis, abundant in artepillin C and CAPE, exhibits potent anti-inflammatory and antioxidant characteristics. Malaysian propolis, rich in flavonoids and terpenes, augments antibacterial and wound-healing efficacy. Chinese propolis, which comprises phenolic alcohols and sesquiterpenes, exhibits cardioprotective and anti-inflammatory properties. Australian propolis, rich in flavonoids and triterpenoids, exhibits antibacterial and anticancer properties. Inflammatory pathways (like NF-κB) are controlled by propolis, which also boosts the immune system, lowers oxidative stress, speeds up glucose metabolism, and kills cancer cells. Its antibacterial activities are efficacious against antibiotic-resistant pathogens.

## Conclusion

In conclusion, propolis is an important resource in integrative medicine and nutrition, with bioactive compounds contributing to its potential in disease prevention, and therapeutic support for several conditions. Whether used raw, as extracts, or with other products, it holds promise in supplements and cosmetics. Further research is needed to address allergies, clarify molecular pathways, and determine appropriate dosages, ensuring its full therapeutic potential is realized.

The main research findings are that Propolis demonstrates various biological actions, encompassing antioxidant, anti-inflammatory, antibacterial, anticancer, antidiabetic, and immunomodulatory properties. Green Brazilian propolis, abundant in artepillin C and CAPE, exhibits potent anti-inflammatory and antioxidant characteristics. Malaysian propolis, rich in flavonoids and terpenes, augments antibacterial and wound-healing efficacy. Chinese propolis, which comprises phenolic alcohols and sesquiterpenes, exhibits cardioprotective and anti-inflammatory properties. Australian propolis, rich in flavonoids and triterpenoids, exhibits antibacterial and anticancer properties. Inflammatory pathways (like NF-κB) are controlled by propolis, which also boosts the immune system, lowers oxidative stress, speeds up glucose metabolism, and kills cancer cells. Its antibacterial activities are efficacious against antibiotic-resistant pathogens.

Future studies should focus on standardizing extraction and formulation methods to reduce compositional variability. Enhancing bioavailability with advanced delivery technologies, like nanotechnology-based encapsulation (e.g., liposomes, nanoparticles) can improve efficacy. Clinical studies are crucial for establishing the optimal dosage, safety, and long-term effects. Understanding the dual antioxidant and pro-oxidant characteristics of propolis will improve its therapeutic use. The medical potential could be enhanced by establishing regulatory standards and investigating its interaction with current treatments.

It should explore the environmental impact on propolis composition and develop sustainable harvesting practices to ensure long-term availability and consistency in therapeutic efficacy. The development of propolis-based products tailored to specific health conditions, combined with an improved understanding of its molecular and cellular mechanisms, will enable the creation of more effective and targeted treatments.

## Supplementary Information

Supplementary material 1.

## Data Availability

Data sharing is not applicable to this article, as no datasets were generated or analysed during the current study; all the articles analysed in this study are cited with its DOI (when available).
