# Supplementary material for: Stingless bee propolis: a comprehensive review of chemical constituents and health efficacy
Source: Nat Prod Bioprospect. 2025 Sep 4;15(1):61. doi: 10.1007/s13659-025-00545-4 (PMC12411399; doi:10.1007/s13659-025-00545-4)
Supplement: Supplementary file 1 — Supplementary material 1. [file 13659_2025_545_MOESM1_ESM.docx]

**Supplementary Material**

**List of compounds have been identified in propolis**

**Table S.1 Other 257 compounds have been identified in propolis**

| **Number** | **Compound Name** | **Structure** | **Bee Species /Botanical Source** | **Identification Methods** |
| --- | --- | --- | --- | --- |
|  | (S)-β-Himachalenes |  | *T. Apicalis* | Q-TOFLC-MS [1] |
|  | Ishwarol |  |  |  |
|  | Leucyl-phenylalanine |  |  |  |
|  | Prolyl-alanyl-lysine |  |  |  |
|  | Ganoderic acid DM |  |  |  |
|  | 1-Hexanol arabinosylglucoside |  |  |  |
|  | N-stearoyl arginine |  |  |  |
|  | N-stearoyl tryptophan |  |  |  |
|  | Dl-threo-1-Phenyl-2-palmitoylamino-3-morpholino-1-propanol |  |  |  |
|  | Acinospesigenin A |  |  |  |
|  | Scopoloside II |  |  |  |
|  | Levocabastine |  |  |  |
|  | Rottlerin |  |  |  |
|  | Oleanolic acid 3-O-beta-d-glucosiduronic acid |  |  |  |
|  | Leucine-aspartate-lysine |  |  |  |
|  | Valine-serine-lysine |  |  |  |
|  | 4-Methoxybenzaldehyde |  | *H. itama*  *G. thoracica*  *T. laeviceps*  *T. biroi* | LC-MS/MS [2] |
|  | Apocynin |  |  |  |
|  | Eupatoriochromene |  |  |  |
|  | Trans-Cinnamaldehyde |  |  |  |
|  | Glabridin |  |  |  |
|  | (-)-8-Prenylnaringenin |  |  |  |
|  | (1R,2R,5S,8R,10R,14R)-20-hydroxy-1,2,14,18,18-pentamethyl-17-oxo-8-(prop-1-en-2-yl) pentacyclohenicosane-5-carboxylic acid |  |  |  |
|  | Abietic acid |  |  |  |
|  | (1R,4aS)-7-(2-Hydroxypropan-2-yl)-1,4a-dimethyl-9-oxo-3,4,10,10a-tetrahydro-2H-phenanthrene-1-carboxylic acid |  | *Friesomelitta doederleini*  *Melipona asilvai*  *Melipona bicolor*  *Melipona fasciculata*  *Melipona flavolineata*  *Melipona marginata*  *Melipona quadrifasciata*  *Melipona quadrifasciata*  *Melipona scutellaris*  *Melipona seminigr*  *Melipona subnitida*  *Plebeia meridionalis*  *Scaptotrigona xanthotricha*  *Tetragona clavipes*  *Tetragonisca angustula*  *Trigona truculenta* | LC-HRMS [3] |
|  | Abietic acid |  |  |  |
|  | 7-Oxodehydroabietic acid |  |  |  |
|  | Cafestol |  |  |  |
|  | 5-[(3Z)-5-Hydroxy-3-methyl-3-penten-1-yl]-1,4a-dimethyl-6-methylenedecahydro-1-naphthalenecarboxylic acid |  |  |  |
|  | (1S,4aR,5S)1,4a-Dimethyl-6-methylene-5-[2-(2-oxo-2,5-dihydro-3-furanyl) ethyl]-decahydro-1-naphthalenecarboxylic acid |  |  |  |
|  | Trans-10-Heptadecenoic acid |  |  |  |
|  | Ethyl myristate |  |  |  |
|  | Trans-δ^2^-11-methyldodecenoic acid |  |  |  |
|  | Docosatrienoic acid |  |  |  |
|  | 9-Oxo-10(E),12(E)-Octadecadienoic acid |  |  |  |
|  | Palmitoleic acid |  |  |  |
|  | Erucamide |  |  |  |
|  | 18-β-Glycyrrhetinic acid |  |  |  |
|  | Urushenol |  |  |  |
|  | 15-Keto-Prostaglandin A_1_ |  |  |  |
|  | 15-Deoxy-δ^12,14^ Prostaglandin |  |  |  |
|  | Sorbicillin |  |  |  |
|  | Bilobol |  |  |  |
|  | 9-cis-Retinal |  |  |  |
|  | Cholest-4-en-3-one |  |  |  |
|  | Longistyline C |  |  |  |
|  | Pyrogallol |  | *Heterotrigona itama* | LC-MS/MS [4] |
|  | Matairesinol |  | *Trigona apicalis* | LC-MS/MS [5] |
|  | Pratensein |  |  |  |
|  | Ursolic acid derivative |  |  |  |
|  | Puerarol |  |  |  |
|  | O-hexanoate |  | Brazilian propolis | UPLC-Q-TOF-MS [6] |
|  | Galangin-5-methyl ether |  |  |  |
|  | Methylbutyrate |  |  |  |
|  | Homopterocarpin |  | Green propolis  Dark propolis | PS/MS  [7] |
|  | Lobelanidin |  | Dark propolis |  |
|  | Scopoloside II |  | Green propolis  Brown propolis |  |
|  | Isorhamnetin |  |  |  |
|  | Quercetin-dimethyl-ether |  | Green propolis |  |
|  | Trihydroxy-dihydrocinnamic acid |  | *Langstroth rational* | HPLC-DAD-ESI-MS/MS  [8] |
|  | Violanthin |  |  |  |
|  | Myricetin-3-O-rhamnoside |  |  |  |
|  | Isorhamnetin-3-O-glucosylgallate |  |  |  |
|  | Prenyl-pentahydroxy-flavone |  |  |  |
|  | Dihydroxy-methoxy chalcone |  |  |  |
|  | Pteron-14-en-7-one |  | Black propolis  Green propolis | GC/EIMS  [9] |
|  | Taraxerone |  |  |  |
|  | α-Amyrin acetate |  |  |  |
|  | β-Amyrin acetate |  |  |  |
|  | Ergosterol |  |  |  |
|  | Quinic acid |  | Black propolis  Green propolis | HPLC-DAD-ESI/MS  [9] |
|  | Dihydroquercetin |  |  |  |
|  | Homovanillic acid |  |  |  |
|  | Tricaffeoylquinic acid |  |  |  |
|  | Feruloyl-caffeoylquinic acid |  |  |  |
|  | Methylkaempferol-O-rutinoside |  |  |  |
|  | Naringenin-C-glucoside |  |  |  |
|  | Apigenin-O-rutinoside |  |  |  |
|  | m-Guaiacol |  | Brazilian red propolis | GC–MS  [10] |
|  | 1-Methoxy-4-(1-propenyl)-benzene |  |  |  |
|  | Methyleugenol |  |  |  |
|  | Methyl o-orsellinate |  |  |  |
|  | Methyl abietate |  |  |  |
|  | Homopterocarpin |  |  |  |
|  | Medicarpin |  |  |  |
|  | 2,4,6-Trimethylphenol |  |  |  |
|  | 4′,7-Dimethoxy-2′-isoflavonol |  |  |  |
|  | Epicatechin |  | Chinese propolis  Brazilian green propolis | RP-HPLC-DAD  [11] |
|  | Morin |  |  |  |
|  | Cinnamyl caffeate |  |  |  |
|  | Baccharin |  |  |  |
|  | Drupanin |  |  |  |
|  | Nemorosone |  | African propolis | LC- HRMS/MS  [12] |
|  | Liquiritigenin |  | African propolis | HRMS/MS [12] |
|  | Formononetin |  |  |  |
|  | Pinocembrin-5-methyl ether |  |  |  |
|  | Bis-methylated quercetin |  | Iraqi propolis | LC-MS [13] |
|  | Pinostrobin |  |  |  |
|  | Hexyl cinnamate |  | Italian propolis | GC−MS  [14] |
|  | Abscisic acid |  | Greek propolis  Chinese propolis | UHPLC-HRMS  [15] |
|  | Ganoderol A |  |  |  |
|  | Imbricatoloic acid |  |  |  |
|  | 3-(13′Z-nonadecenyl)-phenol |  | Cameroonian propolis | GC/MS [16] |
|  | Dicaffeoylquinnic acid |  | Brazilian green propolis (from São Paulo and Minas Gerais) | HPLC-DAD [17] |
|  | 3,5-Diprenyl-4-hydroxycinnamic acid |  |  |  |
|  | Dihydrokaempferol |  | Brazilian green propolis (from Paraná) |  |
|  | 3-Prenyl-4-hydroxycinnamic acid |  |  |  |
|  | 2,2-Dimethyl-2H-1-benzopyran-6-propenoic acid |  |  |  |
|  | 2,2-Dimethyl-8-prenyl-2H-1-benzopyran-6-propenoic acid |  |  |  |
|  | 3-Methoxy-4-hydroxycinnamaldehyde |  | Brazilian brown propolis (from Paraná and Santa Catarina) |  |
|  | Viscidone |  |  |  |
|  | 12-Azabicyclo [9.2.2] pentadeca-1(14),11(15)-dien-13-one |  | Iranian propolis | GC-MS [18] |
|  | Oreophilin |  |  |  |
|  | 3',4'-Dihydro-2'-(morpholin-4-yl)-5',7'-dinitrospiro[cyclopentane-1,3'-quinazoline] |  |  |  |
|  | Dibutyl phthalate |  |  |  |
|  | 2-(2’,4’-Dichloro-phenoxy)phenyl acetic acid |  |  |  |
|  | Margaric acid |  |  |  |
|  | 3-Hydroxy stearic acid |  |  |  |
|  | Eicosanoic acid |  |  |  |
|  | Behenic acid |  |  |  |
|  | Nephrosteranic acid |  |  |  |
|  | Osthole |  |  |  |
|  | Pinostrobin chalcone |  |  |  |
|  | 2’,4’,6’-Trihydroxy chalcone |  |  |  |
|  | 3-Methyl-but-2- enoic acid,2,2- dimethyl-8-oxo-3,4-dihydro-2H,8H- pyrano[3,2- g]chromen-3-yl ester |  |  |  |
|  | 2H-Cyclopentacyclooctene, 4,5,6,7,8,9-hexahydro-1,2,2,3-tetramethyl- |  |  |  |
|  | Germanicol |  |  |  |
|  | 14- Methyl-cholest-7-en-3-ol-15-one |  |  |  |
|  | 4-βH,5α-Eremophil-1 (10)-ene |  | Tehran-Khojir (nearly north of Iran) | GC-MS [19] |
|  | α-Cedrol |  |  |  |
|  | 3,4-Dihydroxybenzaldehyde |  |  |  |
|  | Eicosane |  |  |  |
|  | 2-Nonadecanone |  |  |  |
|  | 3(3-Methoxy, 4-hydroxyphenyl)-2-propenoic acid, methyl ester |  |  |  |
|  | ethyl cinnamate |  |  |  |
|  | Linoleic acid ethyl ether |  |  |  |
|  | Phenylethyl trans-4-coumarate |  |  |  |
|  | Benzyl-trans-4 coumarate |  |  |  |
|  | 3(3-Methoxy, 4-hydroxyphenyl)-2-propenoic acid (isomer 2) |  |  |  |
|  | Isovanillic acid |  |  |  |
|  | 3(3,4-Dimethoxyphenyl)-2-propenoic acid |  |  |  |
|  | Trans-3(4-Hydroxyphenyl)-2-propenoic acid |  |  |  |
|  | Syringic acid |  | *H. itama* | UHPLC-Q-TOF/MS [20] |
|  | Protocatechuic acid |  | Brazilian geopropolis |  |
|  | Vitexin-O-gallate |  |  |  |
|  | Torachrysone-O-(acetyl)- |  |  |  |
|  | Acetyleugenol |  |  |  |
|  | Umbelliferone |  |  |  |
|  | Lapachol |  |  |  |
|  | Mangostin |  |  |  |
|  | Saringosterol |  |  |  |
|  | Stigmasterol |  |  |  |
|  | 24(E)-cycloart-24-ene-26-ol-3-one |  | Brazilian geopropolis | NMR [20] |
|  | Ethyl oleate |  | *Heterotrigona itama (HI)* | GC-MS [21] |
|  | 11-Eicosenoic acid |  |  |  |
|  | Docosanoic acid |  |  |  |
|  | 2,6,10,14,18,22-Tetracosahexaene |  |  |  |
|  | 18-Nonadecen-1-ol |  |  |  |
|  | 5-Heptylresorcinol |  |  |  |
|  | 13,27-Cycloursan-3-one |  |  |  |
|  | Eudesma-4(14),11-diene |  | *Geniotrigona thoracica (GT)* | GC-MS [21] |
|  | Bicyclo[10.8.0]eicosane, cis- |  |  |  |
|  | 13-Docosenamide |  |  |  |
|  | 1-Benzazirene-1- carboxylic acid |  |  |  |
|  | Taraxasterol |  |  |  |
|  | (2S)-5,7-dihydroxy-4′-methoxy-8-prenylflavanone |  | *Tetragonula aff. biroi* | MS and NMR [22] |
|  | (1′S)-2-trans,4-trans-abscisic acid |  |  |  |
|  | 4-(4′-hydroxy-3′-methoxyphenyl)-3,5,7-trihydroxycoumarin |  |  |  |
|  | Anacardic acid |  | *Tetragonula*  *Lepidotrigona*  *Homotrigona* | GC-MS [23] |
|  | Moronic acid |  |  |  |
|  | Ursolic acid |  |  |  |
|  | Lantanolic aci |  |  |  |
|  | Erythrodiol |  |  |  |
|  | Cycloartenone |  |  |  |
|  | Lupenone |  |  |  |
|  | 3-Isomangostin |  | *T. pagdeni* | HPLC [24] |
|  | γ-Mangostin |  |  |  |
|  | β-Mangostin |  |  |  |
|  | α-Mangostin |  |  |  |
|  | Kaurenal |  | Mexicana propolis | GC-MS [25] |
|  | Kaurenoic acid |  |  |  |
|  | Hydroxyphenyl acetic  Acid |  |  |  |
|  | Cardol C17:2 |  |  |  |
|  | Dihydrocubebin |  |  |  |
|  | β-Amyrenone |  |  |  |
|  | Ethylamine |  |  |  |
|  | 3,4-Methylenedioxy  secoisolariciresinol |  |  |  |
|  | Pinitol |  |  |  |
|  | Arabitol |  |  |  |
|  | Glycerol |  |  |  |
|  | 2-Trans-abscisate |  | *Tetragonula biroi* | LC-MS and HPLC-UV-ELSD [26] |
|  | 3-Dehydrocholate |  |  |  |
|  | Glycyrrhetinate |  |  |  |
|  | Broussoflavonol F |  | *Tetragonula aff. biroi* | HPLC [27] |
|  | 2',3'-Dihydro-3'-hydroxypapuanic acid |  |  |  |
|  | (1'S)-2-cis,4-trans-abscisic acid |  |  |  |
|  | (2S)-5,7-dihydroxy-4'-methoxy-8-prenylflavanone |  |  |  |
|  | Lupeol |  | *Lisotrigona cacciae* | GC-MS [28] |
|  | 3-Geranyloxy-1,7-dihydroxyxanthone |  |  |  |
|  | 7-Geranyloxy-1,3-dihydroxyxanthone |  |  |  |
|  | δ-Tocotrienol |  |  |  |
|  | Androsta-1,4-dien-3-one,17- |  | *Trigona apicalis* | GC-MS [29] |
|  | 9-Isopropyl-1-methyl-2-methylene-5-oxatricydo[5.4.0.0(3,8)]undecane |  |  |  |
|  | (S)-3'-hydroxy-4-methoxydalbergione |  | Nepalese propolis | LC-CD [30] |
|  | (S)-4-methoxydalbergione |  |  |  |
|  | 1-(3',4'-Dihydroxy-2'-methoxyphenyl)-3-(phenyl)propane |  | Mexican red propolis | 2D-NMR and ESI-MS/MS  [31] |
|  | (Z)-1-(2'-methoxy-4',5'dihydroxyphenyl)-2-(3-phenyl)propene |  |  |  |
|  | 3,10-Dihydroxy-9-methoxypterocarpan |  | Brazilian red propolis | TLC and NMR [32] |
|  | 6a-Ethoxymedicarpin |  |  |  |
|  | Biochanin A |  |  |  |
|  | (3*S*)-Ferreirin |  |  |  |
|  | (3*S*)-Vestitone |  |  |  |
|  | (3*S*)-Mucronulatol |  |  |  |
|  | (3*S*)-Isovestitol |  |  |  |
|  | (6a*R*,11a*R*)-4-Methoxymedicarpin |  |  |  |
|  | Neovestitol |  | Cuban propolis | GC-MS [33] |
|  | (3*S*)-Mucronulatol |  |  |  |
|  | 2',6',4-Tryhydroxy-4'-methoxydihydrochalcone |  | Canadian propolis | GC-MS [34] |
|  | 2',6'-Dihydroxy-4',4-dimethoxydihydrochalcone |  |  |  |
|  | Odoratin |  | Nepalese propolis (from Chitwan) | GC-MS [35] |
|  | 5-Methoxy-3-hidroxyflavanone |  | Propolis from Northeast of Portugal | HPLC [36] |
|  | 5,7,3',4'-Tetrahydroxy-5'-*C*-geranylflavanone |  | Japanese propolis (from Okinawa) | MS and 2D-NMR [37] |
|  | 5,7,3',4'-Tetrahydroxy-6-*C*-geranylflavanone |  |  |  |
|  | 7-O-Prenylpinocembrin |  | Greek propolis | 2D-NMR and MS [38] |
|  | 7-O-Prenylstrobopinin |  |  |  |
|  | Macarangin |  | Kenyan propolis (from Mwingi) | NMR [39] |
|  | 2'-(8"-Hydroxy-3",8"-dimethyl-oct-2"-enyl)-quercetin |  | Propolis from  Solomon Islands | MS and AD-NMR [40] |
|  | 8-(8"-Hydroxy-3",8"-dimethyl-oct-2"-enyl)-quercetin |  |  |  |
|  | Hexamethoxy flavone |  | Egyptian propolis | GC-MS [41] |
|  | 6-Cinnamylchrysin |  | Chinese Propolis | NMR [42] |
|  | Luteolin |  | Chinese Propolis | HPCE-UV [43] |
|  | Retusapurpurin A |  | Brazilian red propolis | ESI/MS and HPLC-PDA-ESI/MS [44,45] |
|  | Daidzein |  | Brazilian red propolis | RP-HPLC [10] |
|  | Elemicin |  | Brazilian red propolis | GC-MS [45] |
|  | Guttiferone E |  | Cuban and Brazilian red propolis | HPLC-PDA-ESI/MS [46,45] |
|  | Xanthochymol |  |  |  |
|  | Isoliquiritigenin |  | Cuban and Brazilian propolis | HPLC-PDA-ESI/MS, GC/MS, NMR and HPLC [46,47] |
|  | (3α,4α)- 4- Methyl- stigmast-22-en-3- ol |  | Iranian Propolis | GC-MS [48] |
|  | Artepillin C |  | *B. dracunculifolia* | RPHPLC [49] |
|  | Hentriacontene |  | Yemeni propolis | GC-MS [50] |
|  | Triacontene |  |  |  |
|  | Moretenol |  |  |  |
|  | Pentatriacontene |  |  |  |
|  | Dammaradienyl pentanoate |  |  |  |
|  | Heneicosane |  |  |  |
|  | α-Amyryl pentanoate |  |  |  |
|  | Benzyl alcohol |  | Polish propolis | GC-MS [51] |
|  | Hydroquinone |  |  |  |
|  | Coniferyl benzoate |  |  |  |
|  | Benzyl *p*-coumarate (*E*) |  |  |  |
|  | Phenylethyl caffeate |  |  |  |
|  | Alpinon |  |  |  |
|  | Isosakuranetin |  |  |  |
|  | Agathic acid |  | Brazilian red, and green propolis ( from São Paulo)  Brazilian yellow propolis  (from Mato Grosso do Sul) | UPLC-ESI(−)-MS/MS [52] |
|  | 3-Prenyl-4-dihydrocinnamoyloxy cinnamic acid |  |  |  |
|  | 3,5-DI-O-caffeoylquinic acid |  |  |  |
|  | Betuletol |  |  |  |

**References**

1. MOHAMED WAS, ISMAIL NZ, MUHAMAD M, et al. Q-tof lc-ms compounds evaluation of propolis extract derived from malaysian stingless bees, tetrigona apicalis, and their bioactivities in breast cancer cell, mcf7. Saudi journal of biological sciences*,* 2022; 29(10): 103403; <https://doi.org/10.1016/j.sjbs.2022.103403>.

2. PRATAMI DK, SAHLAN M, BAYU A, et al. Characteristics of indonesian stingless bee propolis and study of metabolomic properties based on region and species. Molecules*,* 2024; 29(17): 4037; <https://doi.org/10.3390/molecules29174037>.

3. TURCO JF, MOKOCHINSKI JB, TORRES YR. Lipidomic analysis of geopropolis of brazilian stingless bees by lc-hrms. Food Research International*,* 2023; 167: 112640; <https://doi.org/10.1016/j.foodres.2023.112640>.

4. MOHAN S, AZMI WA, SANTHANAM R, et al. Photoprotective properties of four structure propolis from heterotrigona itama stingless beehive: Fractionation, bioactivity analysis, and chemical profiling. Heliyon*,* 2024; 10(20); <https://doi.org/10.1016/j.heliyon.2024.e39164>.

5. MOHAMED WAS, ISMAIL NZ, OMAR EA, et al. Gc‐ms evaluation, antioxidant content, and cytotoxic activity of propolis extract from peninsular malaysian stingless bees, tetrigona apicalis. Evidence‐Based Complementary and Alternative Medicine*,* 2020; 2020(1): 8895262; <https://doi.org/10.1155/2020/8895262>.

6. FERNANDES-SILVA CC, SALATINO A, SALATINO MLF, et al. Chemical profiling of six samples of brazilian propolis. Química Nova*,* 2013; 36: 237-240; <http://dx.doi.org/10.1590/S0100-40422013000200006>.

7. VIEIRA ALS, CORREIA VTDV, RAMOS ALCC, et al. Evaluation of the chemical profile and antioxidant capacity of green, brown, and dark propolis. Plants*,* 2023; 12(18): 3204; <https://doi.org/10.3390/plants12183204>.

8. FERREIRA JM, FERNANDES‐SILVA CC, SALATINO A, et al. New propolis type from north‐east brazil: Chemical composition, antioxidant activity and botanical origin. Journal of the Science of Food and Agriculture*,* 2017; 97(11): 3552-3558; <https://doi.org/10.1002/jsfa.8210>.

9. RIGHI AA, NEGRI G, SALATINO A. Comparative chemistry of propolis from eight brazilian localities. Evidence‐Based Complementary and Alternative Medicine*,* 2013; 2013(1): 267878; <https://doi.org/10.1155/2013/267878>.

10. ALENCAR SMD, OLDONI TC, CASTRO M, et al. Chemical composition and biological activity of a new type of brazilian propolis: Red propolis. Journal of Ethnopharmacology*,* 2007; 113(2): 278-283; <https://doi.org/10.1016/j.jep.2007.06.005>.

11. SUN S, HE J, LIU M, et al. A great concern regarding the authenticity identification and quality control of chinese propolis and brazilian green propolis. J. Food Nutr. Res*,* 2019; 7(10): 725-735; <http://dx.doi.org/10.12691/jfnr-7-10-6>.

12. ZHANG T, OMAR R, SIHERI W, et al. Chromatographic analysis with different detectors in the chemical characterisation and dereplication of african propolis. Talanta*,* 2014; 120: 181-190; <https://doi.org/10.1016/j.talanta.2013.11.094>.

13. SULAIMAN GM, AL SAMMARRAE KW, AD’HIAH AH, et al. Chemical characterization of iraqi propolis samples and assessing their antioxidant potentials. Food and Chemical Toxicology*,* 2011; 49(9): 2415-2421; <https://doi.org/10.1016/j.fct.2011.06.060>.

14. ALIBONI A, D’ANDREA A, MASSANISSO P. Propolis specimens from different locations of central italy: Chemical profiling and gas chromatography− mass spectrometry (gc− ms) quantitative analysis of the allergenic esters benzyl cinnamate and benzyl salicylate. Journal of agricultural and food chemistry*,* 2011; 59(1): 282-288; <https://doi.org/10.1021/jf1034866>.

15. STAVROPOULOU M-I, TERMENTZI A, KASIOTIS KM, et al. Untargeted ultrahigh-performance liquid chromatography-hybrid quadrupole-orbitrap mass spectrometry (uhplc-hrms) metabolomics reveals propolis markers of greek and chinese origin. Molecules*,* 2021; 26(2): 456; <https://doi.org/10.3390/molecules26020456>.

16. KARDAR M, ZHANG T, COXON G, et al. Characterisation of triterpenes and new phenolic lipids in cameroonian propolis. Phytochemistry*,* 2014; 106: 156-163; <https://doi.org/10.1016/j.phytochem.2014.07.016>.

17. SAWAYA ACHF, BARBOSA DA SILVA CUNHA I, MARCUCCI MC. Analytical methods applied to diverse types of brazilian propolis. Chemistry Central Journal*,* 2011; 5: 1-10; <https://doi.org/10.1186/1752-153x-5-27>.

18. AFROUZAN H, TAHGHIGHI A, ZAKERI S, et al. Chemical composition and antimicrobial activities of iranian propolis. Iran Biomed J*,* 2018; 22(1): 50-65; <http://dx.doi.org/10.22034/ibj.22.1.50>.

19. MOHAMMADZADEH S, SHARIATPANAHI M, HAMEDI M, et al. Chemical composition, oral toxicity and antimicrobial activity of iranian propolis. Food chemistry*,* 2007; 103(4): 1097-1103; <https://doi.org/10.1016/j.foodchem.2006.10.006>

20. ZHAO L, YU M, SUN M, et al. Rapid determination of major compounds in the ethanol extract of geopropolis from malaysian stingless bees, heterotrigona itama, by uhplc-q-tof/ms and nmr. Molecules*,* 2017; 22(11): 1935; <https://doi.org/10.3390/molecules22111935>

21. IBRAHIM N, ZAKARIA AJ, ISMAIL Z, et al. Application of gcms and ftir fingerprinting in discriminating two species of malaysian stingless bees propolis. International Journal of Engineering & Technology*,* 2018; 7(4.43): 106-112; <https://doi.org/10.14419/ijet.v7i4.43.25828>.

22. MIYATA R, SAHLAN M, ISHIKAWA Y, et al. Propolis components from stingless bees collected on south sulawesi, indonesia, and their xanthine oxidase inhibitory activity. Journal of natural products*,* 2019; 82(2): 205-210; <https://doi.org/10.1021/acs.jnatprod.8b00541.s003>.

23. POPOVA M, TRUSHEVA B, CHIMSHIROVA R, et al. Chemical profile and antioxidant capacity of propolis from tetragonula, lepidotrigona, lisotrigona and homotrigona stingless bee species in vietnam. Molecules*,* 2022; 27(22): 7834; <https://doi.org/10.3390/molecules27227834>

24. KONGKIATPAIBOON S, VONGSAK B, MACHANA S, et al. Simultaneous hplc quantitative analysis of mangostin derivatives in tetragonula pagdeni propolis extracts. Journal of King Saud University-Science*,* 2016; 28(2): 131-135; <https://doi.org/10.1016/j.jksus.2015.06.007>

25. GERGINOVA D, POPOVA M, CHIMSHIROVA R, et al. The chemical composition of scaptotrigona mexicana honey and propolis collected in two locations: Similarities and differences. Foods*,* 2023; 12(17): 3317; <https://doi.org/10.3390/foods12173317>.

26. ALANAZI S. Antineoplastic and antitrypanosomal properties of propolis from tetragonula biroi friese. Molecules*,* 2022; 27(21): 7463; <https://doi.org/10.3390/molecules27217463>.

27. MIYATA R, SAHLAN M, ISHIKAWA Y, et al. Propolis components and biological activities from stingless bees collected on south sulawesi, indonesia. HAYATI Journal of Biosciences*,* 2020; 27(1): 82-82; <https://doi.org/10.4308/hjb.27.1.82>.

28. GEORGIEVA K, POPOVA M, DIMITROVA L, et al. Phytochemical analysis of vietnamese propolis produced by the stingless bee lisotrigona cacciae. PLoS One*,* 2019; 14(4): e0216074; <https://doi.org/10.1371/journal.pone.0216074>.

29. GAPAR NAA, ASEM N, ABD HAPIT NH, et al. Phytochemical profiling and anticancer activities of ethanolic and aqueous propolis extract of trigona apicalis.: Received 2023-01-12; accepted 2023-02-27; published 2023-06-06. Journal of Health and Translational Medicine (JUMMEC)*,* 2023: 141-148; <https://doi.org/10.22452/jummec.sp2023no1.13>

30. SHRESTHA SP, NARUKAWA Y, TAKEDA T. Chemical constituents of nepalese propolis: Isolation of new dalbergiones and related compounds. Journal of Natural Medicines*,* 2007; 61: 73-76; <https://doi.org/10.1007/s11418-006-0024-8>.

31. LOTTI C, CAMPO FERNANDEZ M, PICCINELLI AL, et al. Chemical constituents of red mexican propolis. Journal of agricultural and food chemistry*,* 2010; 58(4): 2209-2213; <https://doi.org/10.1021/jf100070w>.

32. LI F, AWALE S, TEZUKA Y, et al. Cytotoxic constituents from brazilian red propolis and their structure–activity relationship. Bioorganic & Medicinal Chemistry*,* 2008; 16(10): 5434-5440; <https://doi.org/10.1016/j.bmc.2008.04.016>.

33. CAMPO FERNÁNDEZ M, CUESTA-RUBIO O, ROSADO PEREZ AS, et al. Gc-ms determination of isoflavonoids in seven red cuban propolis samples. Journal of agricultural and food chemistry*,* 2008; 56(21): 9927-9932; <https://doi.org/10.1021/jf801870f>.

34. CHRISTOV R, TRUSHEVA B, POPOVA M, et al. Chemical composition of propolis from canada, its antiradical activity and plant origin. Natural Product Research*,* 2006; 20(06): 531-536; <https://doi.org/10.1080/14786410500056918>.

35. SHRESTHA SP, NARUKAWA Y, TAKEDA T. Chemical constituents of nepalese propolis (ii). Chemical and pharmaceutical bulletin*,* 2007; 55(6): 926-929; <https://doi.org/10.1002/chin.200748207>.

36. FALCÃO SI, VILAS-BOAS M, ESTEVINHO LM, et al. Phenolic characterization of northeast portuguese propolis: Usual and unusual compounds. Analytical and bioanalytical chemistry*,* 2010; 396: 887-897; <https://doi.org/10.1007/s00216-009-3232-8>.

37. KUMAZAWA S, GOTO H, HAMASAKA T, et al. A new prenylated flavonoid from propolis collected in okinawa, japan. Bioscience, biotechnology, and biochemistry*,* 2004; 68(1): 260-262; <https://doi.org/10.1271/bbb.68.260>.

38. MELLIOU E, CHINOU I. Chemical analysis and antimicrobial activity of greek propolis. Planta medica*,* 2004; 70(06): 515-519; <https://doi.org/10.1055/s-2004-827150>

39. PETROVA A, POPOVA M, KUZMANOVA C, et al. New biologically active compounds from kenyan propolis. Fitoterapia*,* 2010; 81(6): 509-514; <https://doi.org/10.1016/j.fitote.2010.01.007>.

40. INUI S, SHIMAMURA Y, MASUDA S, et al. A new prenylflavonoid isolated from propolis collected in the solomon islands. Bioscience, biotechnology, and biochemistry*,* 2012; 76(5): 1038-1040; <https://doi.org/10.1271/bbb.120021>.

41. HEGAZI AG, EL HADY FKA. Egyptian propolis: 3. Antioxidant, antimicrobial activities and chemical composition of propolis from reclaimed lands. Zeitschrift für Naturforschung C*,* 2002; 57(3-4): 395-402; <https://doi.org/10.1515/znc-2002-3-432>.

42. USIA T, BANSKOTA AH, TEZUKA Y, et al. Constituents of chinese propolis and their antiproliferative activities. Journal of natural products*,* 2002; 65(5): 673-676; <https://doi.org/10.1021/np010486c>.

43. CAO Y, WANG Y, YUAN Q. Analysis of flavonoids and phenolic acid in propolis by capillary electrophoresis. Chromatographia*,* 2004; 59(1): 135-140; <https://doi.org/10.1365/s10337-003-0138-z>

44. DA SILVA FROZZA CO, GARCIA CSC, GAMBATO G, et al. Chemical characterization, antioxidant and cytotoxic activities of brazilian red propolis. Food and Chemical Toxicology*,* 2013; 52: 137-142; <https://doi.org/10.1016/j.fct.2012.11.013>.

45. TRUSHEVA B, POPOVA M, BANKOVA V, et al. Bioactive constituents of brazilian red propolis. Evidence‐Based Complementary and Alternative Medicine*,* 2006; 3(2): 249-254; <https://doi.org/10.1093/ecam/nel006>.

46. PICCINELLI AL, LOTTI C, CAMPONE L, et al. Cuban and brazilian red propolis: Botanical origin and comparative analysis by high-performance liquid chromatography–photodiode array detection/electrospray ionization tandem mass spectrometry. Journal of agricultural and food chemistry*,* 2011; 59(12): 6484-6491; <https://doi.org/10.1021/jf201280z>

47. OLDONI TLC, CABRAL IS, D’ARCE MAR, et al. Isolation and analysis of bioactive isoflavonoids and chalcone from a new type of brazilian propolis. Separation and purification Technology*,* 2011; 77(2): 208-213; <https://doi.org/10.1016/j.seppur.2010.12.007>.

48. AHANGARI Z, NASERI M, VATANDOOST F. Propolis: Chemical composition and its applications in endodontics. Iranian endodontic journal*,* 2018; 13(3): 285; <https://doi.org/10.22037/iej.v13i3.20994>.

49. PARK YK, PAREDES-GUZMAN JF, AGUIAR CL, et al. Chemical constituents in baccharis dracunculifolia as the main botanical origin of southeastern brazilian propolis. Journal of agricultural and food chemistry*,* 2004; 52(5): 1100-1103; <https://doi.org/10.1021/jf021060m>.

50. AL-GHAMDI AA, BAYAQOOB NI, RUSHDI AI, et al. Chemical compositions and characteristics of organic compounds in propolis from yemen. Saudi journal of biological sciences*,* 2017; 24(5): 1094-1103; <https://doi.org/10.1016/j.sjbs.2016.12.012>.

51. POPOVA M, GIANNOPOULOU E, SKALICKA-WOŹNIAK K, et al. Characterization and biological evaluation of propolis from poland. Molecules*,* 2017; 22(7): 1159; <https://doi.org/10.3390/molecules22071159>.

52. MACHADO CS, MOKOCHINSKI JB, DE LIRA TO, et al. Comparative study of chemical composition and biological activity of yellow, green, brown, and red brazilian propolis. Evid Based Complement Alternat Med*,* 2016; 2016: 6057650; <https://doi.org/10.1155/2016/6057650>.
